# Syntheses, Crystal Structures, and Antitumor Activities of Copper(II) and Nickel(II) Complexes with 2-((2-(Pyridin-2-yl)hydrazono)methyl)quinolin-8-ol

**DOI:** 10.3390/ijms19071874

**Published:** 2018-06-26

**Authors:** Qi-Yuan Yang, Qian-Qian Cao, Qi-Pin Qin, Cai-Xing Deng, Hong Liang, Zhen-Feng Chen

**Affiliations:** State Key Laboratory for Chemistry and Molecular Engineering of Medicinal Resources, School of Chemistry and Pharmacy, Guangxi Normal University, 15 Yucai Road, Guilin 541004, China; 2016110029@stu.gxnu.edu.cn (Q.-Y.Y.); 2014011049@stu.gxnu.edu.cn (Q.-Q.C.);2014110004@stu.gxnu.edu.cn (Q.-P.Q.); 2016010981@stu.gxnu.edu.cn (C.-X.D.); hliang@gxnu.edu.cn (H.L.)

**Keywords:** quinolinyl hydrazine, copper(II) complex, cytotoxicity, apoptosis

## Abstract

Two transition metal complexes with 2-((2-(pyridin-2-yl)hydrazono)methyl)quinolin-8-ol (L), [Cu(L)Cl_2_]_2_ (**1**) and [Ni(L)Cl_2_]·CH_2_Cl_2_ (**2**), were synthesized and fully characterized. Complex **1** exhibited high in vitro antitumor activity against SK-OV-3, MGC80-3 and HeLa cells with IC_50_ values of 3.69 ± 0.16, 2.60 ± 0.17, and 3.62 ± 0.12 μM, respectively. In addition, complex **1** caused cell arrest in the S phase, which led to the down-regulation of Cdc25 A, Cyclin B, Cyclin A, and CDK2, and the up-regulation of p27, p21, and p53 proteins in MGC80-3 cells. Complex **1** induced MGC80-3 cell apoptosis via a mitochondrial dysfunction pathway, as shown by the significantly decreased level of bcl-2 protein and the loss of Δψ, as well as increased levels of reactive oxygen species (ROS), intracellular Ca^2+^, cytochrome C, apaf-1, caspase-3, and caspase-9 proteins in MGC80-3 cells.

## 1. Introduction

Numerous platinum(II) complexes have been successfully used for the treatment of different types of cancers [1]. Platinum complexes stand out among chemotherapeutic agents for its high efficacy in combination therapy. However, they also show drawbacks like toxicity and drug resistance [2]. Especially, the clinical use of cisplatin is severely limited by its unwanted side effects, including ototoxicity and nephrotoxicity, which reduce patient tolerance during treatment and interfere with the long-term quality of life [3]. Therefore, it is necessary to explore other nonplatinum complexes that could offer high efficacy with fewer side effects.

Many studies show that the copper and nickel complexes play an important role in the endogenous oxidative DNA damage associated with aging and cancer [4,5,6,7,8]. For example, complexes with Cu(II) ion show high DNA binding and DNA cleavage activities [9], and copper complexes induced reversible condensation of DNA and apoptosis in osteosarcoma MG-63 cell lines [10]. Many nickel complexes bearing biological activity have been reported including Ni(II) complexes with antitumor activity [11,12]. Nickel complexation with lidocaine enhances the DNA binding affinity, DNA cleavage activity, and cytotoxic properties of lidocaine [13]; Nickel complexes also show considerable cytotoxic activity against the human hepatocarcinoma cells (Hep-G2), human leukemic cells (HL-60), and human prostatic carcinoma cells (PC-3) [14]. Therefore, the synthesis and biological testing of copper and nickel complexes have become an important area of current bioinorganic chemistry research [15,16,17].

**Scheme 1:**
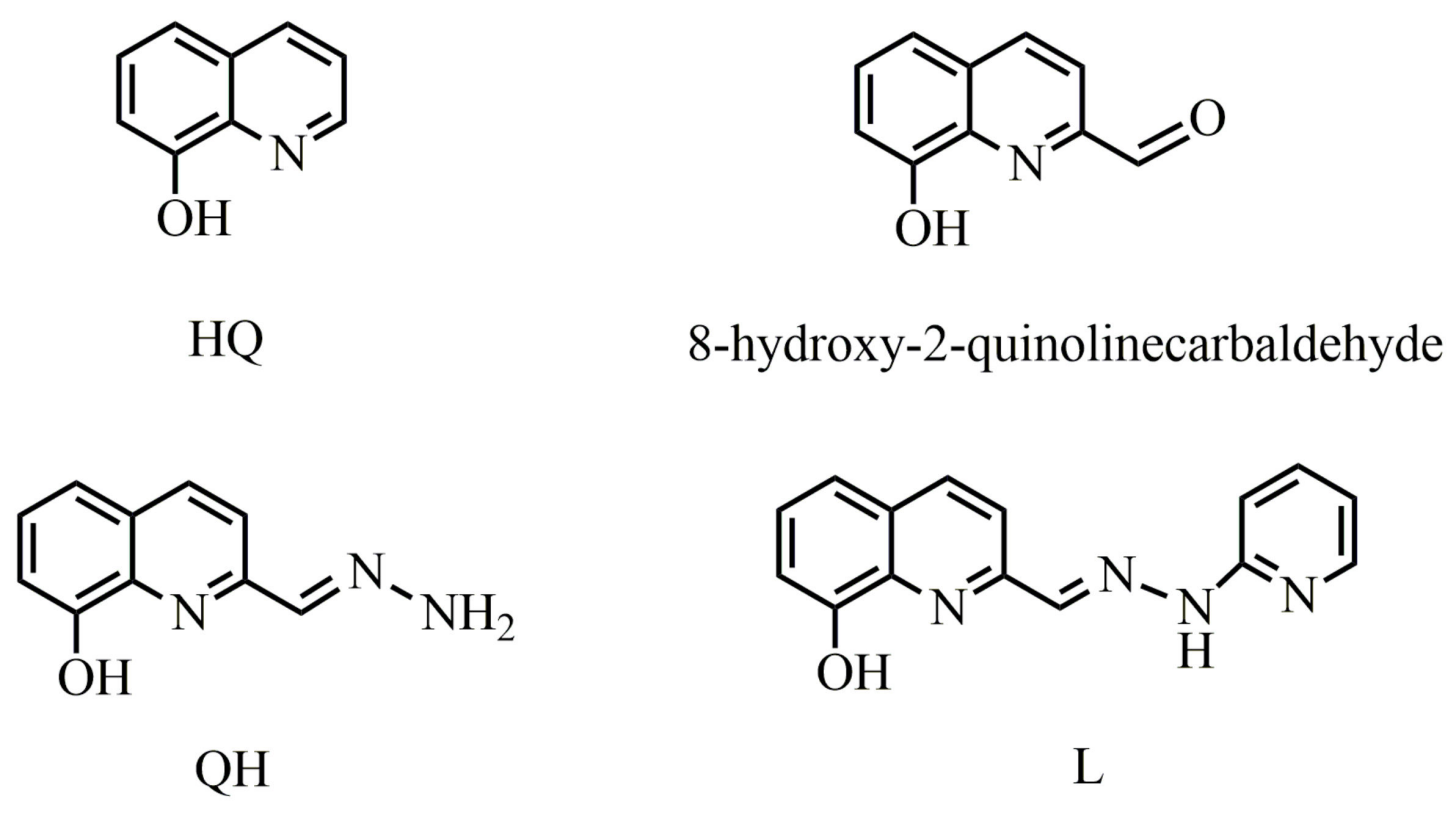
The structures of 8-hydroxyquinoline (HQ), quinolinyl hydrazine (QH) and L.

The compound 8-Hydroxyquinoline (HQ) has attracted considerable interest as a privileged structure (Scheme 1), and 8-hydroxyquinoline derivatives (HQs) have been explored for a broad range of biological applications [18], such as metal-chelators for neuroprotection, chelators of metalloproteins, inhibitors of 2OG-dependent enzymes, *Mycobacterium tuberculosis* inhibitors, botulinum neurotoxin inhibitors and anticancer, anti-HIV, antifungal, antileishmanial, and antischistosomal agents [19,20,21]. The HQs with anticancer or anti-Alzheimer activities include mainly halogenated derivatives [22,23], diperazino and alkyno derivatives [24,25], nitro derivatives [26,27,28], carboxylic and carboxamido derivatives [29,30,31], amino and imino derivatives [32,33], sulfoxine and sulfonamide derivatives [34,35,36], Bis- and poly-HQs [37,38], HQ bioconjugates [39,40,41], and other HQ derivatives [42]. In addition, it is well known that quinolinylhydrazones show various important biological activities and the quinoline ring plays an important role in the development of new anticancer agents [43,44,45,46,47]. For example, the quinolinylhydrazones exhibit significant cytotoxicity in comparison with similar reported systems and the apoptosis induction in MCF-7 cancer cells increased when it was coordinated with the gold nanoparticle surface [48]. Recently, the synthesis of 2-((2-(pyridin-2-yl)hydrazono)methyl)quinolin-8-ol (L) was reported [49]. The metal complexes of HQs show enhanced tumor cytotoxicity [50,51,52,53,54,55,56], including ruthenium [50,51], gold [52], platinum [53], copper [43,48,49], and vanadium [44] complexes. However, there are few reports on the synthesis and antitumor activity of Cu(II) and Ni(II) complexes. Chan et al. found that 8-hydroxy-2-quinolinecarbaldehyde (Scheme 1) showed the highest in vitro cytotoxicity against the human cancer cell lines, including MDA231, T-47D, Hs578t, SaoS2, K562, SKHep1, and Hep3B [42].

Therefore, as part of our continuing work on the synthesis, characterization and medicinal application of metal complexes with HQ [45,46,47], we report the synthesis and characterization of Cu(II) and Ni(II) complexes with 2-((2-(pyridin-2-yl)hydrazono)methyl)quinolin-8-ol (L) and the in vitro cytotoxicities against seven tumor cells and their antitumor mechanism.

## 2. Results

### 2.1. Synthesis

**Scheme 2:**
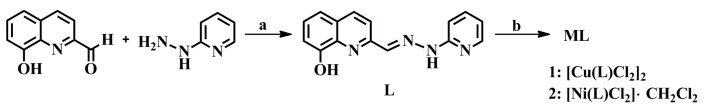
The synthetic routes for ligand (L) and its metal complexes **1** and **2**. Reagents are as follows: (**a**) EtOH, r.t, 16 h; (**b**) CuCl_2_ or NiCl_2_, MeOH/CH_2_Cl_2_.

As outlined in Scheme 2, complexes **1**, **2** were synthesized by the reaction of L with CuCl_2_·2H_2_O and NiCl_2_·6H_2_O in hot methanol, respectively. They were satisfactorily characterized by mass spectrometry (MS), elemental analysis (EA), infrared spectroscopy (IR), and single-crystal X-ray diffraction analysis. The absorptions around 1550–1650 cm^−1^ of the IR (Figures S3–S5) were assigned to the imine bond stretching vibrations of L. The imine bonds of complexes **1** and **2** underwent a left-shift of 10–60 cm^−1^ upon coordination, indicating the participation of this group in coordination. The single-crystal structure analysis suggested that the Cu(II) complex was [Cu(L)Cl_2_]_2_ (**1**) and the Ni(II) complex was [Ni(L)Cl_2_]·CH_2_Cl_2_ (**2**).

### 2.2. Crystal Structures of Complexes **1** and **2**

**Figure 1:**
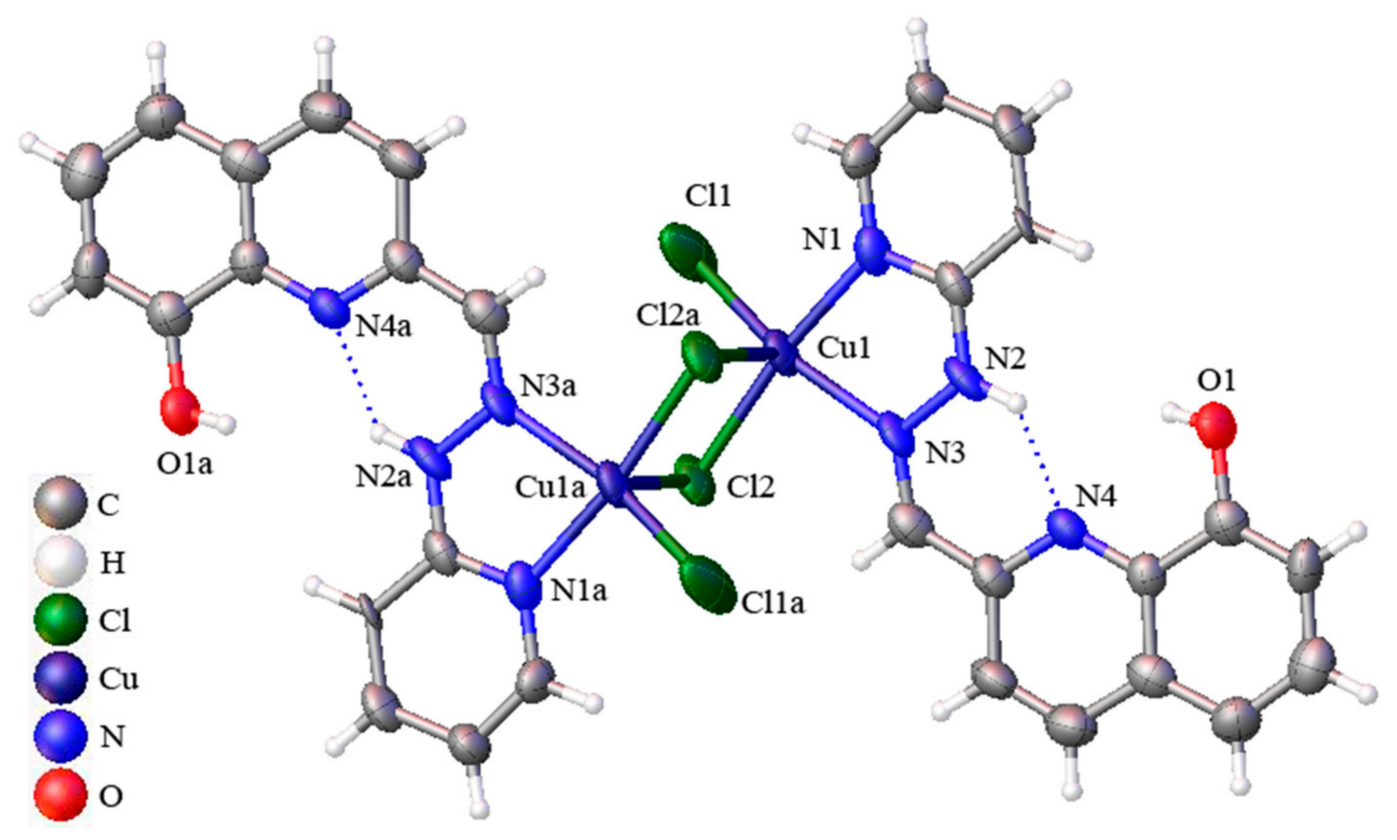
The crystal structure of Cu(II) complex **1**.

**Figure 2:**
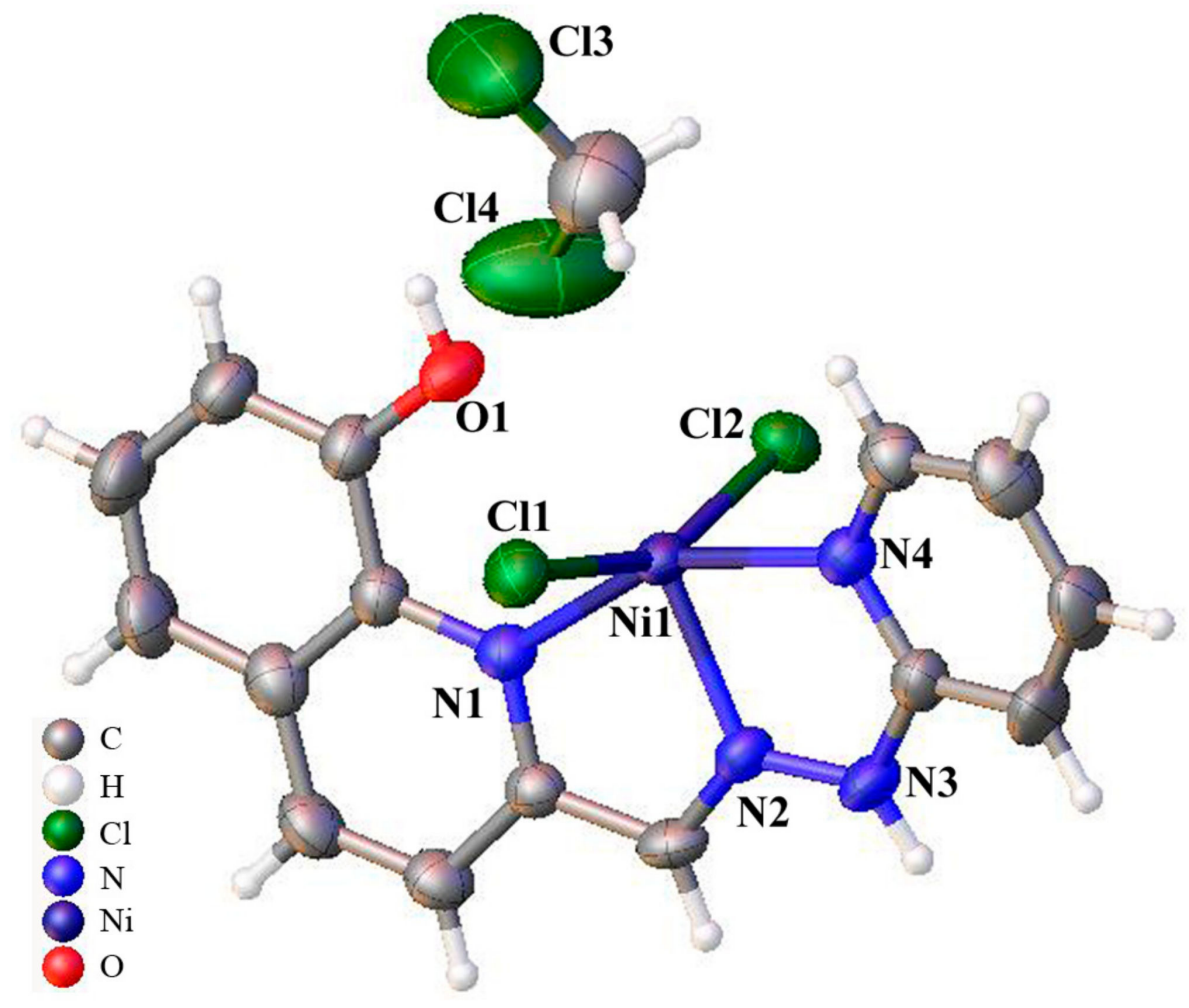
The crystal structure of complex **2**.

The crystal data and refinement details of complexes **1** and **2** are summarized in Table S1 (Supporting Information), and the selected bond lengths and angles are listed in Tables S2 and S3. The crystal structures of complexes **1** and **2** are shown in Figure 1 and Figure 2. Complexes **1** and **2** have different coordination pattern. Complex **1** was a dinuclear L-Cu-Cl-(μ-Cl)_2_-Cu-Cl-L complex, and the Cu(II) ions were coordinated by three Cl and two N atoms from L in a distorted square pyramidal geometry.

In complex **2**, the central Ni^II^ adopted an approximately five-coordinated tetragonal pyramidal geometry.

### 2.3. Stability in Solution

Ligand L, complexes **1** and **2** were tested for their stabilities in both dimethyl sulfoxide (DMSO) and Tris-HCl buffer solution (TBS) (TBS solution with pH at 7.35, containing 1% DMSO) by means of UV-Vis spectroscopy. The time-dependent (in the time course of 0, 12, 24, 36 and 48 h) UV-Vis spectra of each complex dissolved in TBS solution are shown in Figure S1. There were no obvious changes in the spectral characteristics and the peak absorptions for ligand L, complexes **1** and **2** over the time course. In addition, the stabilities of L, complexes **1** and **2** were monitored by high performance liquid chromatography (HPLC) detected at 245 nm, and no significant change was observed for these three compounds in TBS at 0, 24, and 48 h (Figure S2). Combining the ESI-MS data, the results suggested that complex **2** was stable in TBS solution, and complexes **1** was stable in TBS solution as mononuclear species because it was dissociated in water solution and Tris-HCl buffer (see the results of Figure S9).

### 2.4. In Vitro Cytotoxicity

The in vitro cytotoxicities of L, complexes **1** and **2** were evaluated by MTT assay in seven human tumor cell lines Hep-G2, SK-OV-3, MGC80-3, HeLa, T-24, BEL-7402, and NCI-H460 and one normal liver cell line HL-7702. Each compound was prepared as 2.0 mM DMSO stock solution before it was diluted in PBS buffer to 20 μM aqueous solutions (containing 2.5% DMSO). These 20 μM aqueous solutions were stable and no precipitate was formed.

The in vitro antitumor activities of complex **1** were further evaluated by determining the corresponding IC_50_ values. As shown in Table 1, the IC_50_ values of complex **1** against SK-OV-3, MGC80-3 and HeLa were 3.69 ± 0.16, 2.60 ± 0.17, and 3.62 ± 0.12 μM, respectively, which were approximately 11.6, 15.6, and 16.2 fold increases compared with that of the free L. In addition, complex **1** exhibited stronger cytotoxicities than cisplatin towards the SK-OV-3, MGC80-3, and HeLa tumor cells. In summary, complex **1** exhibited a lower IC_50_ value for MGC80-3 cells than other cells and higher cytotoxicity than complex **2**. Thus, complex **1** was chosen to study the underlying cellular and molecular mechanisms of its cytotoxicity. (As a support material, Inhibitory rates (%) of compounds were shown in Table S4)

### 2.5. Cell Cycle Analysis and Expressions of the Related Proteins

**Figure 3:**
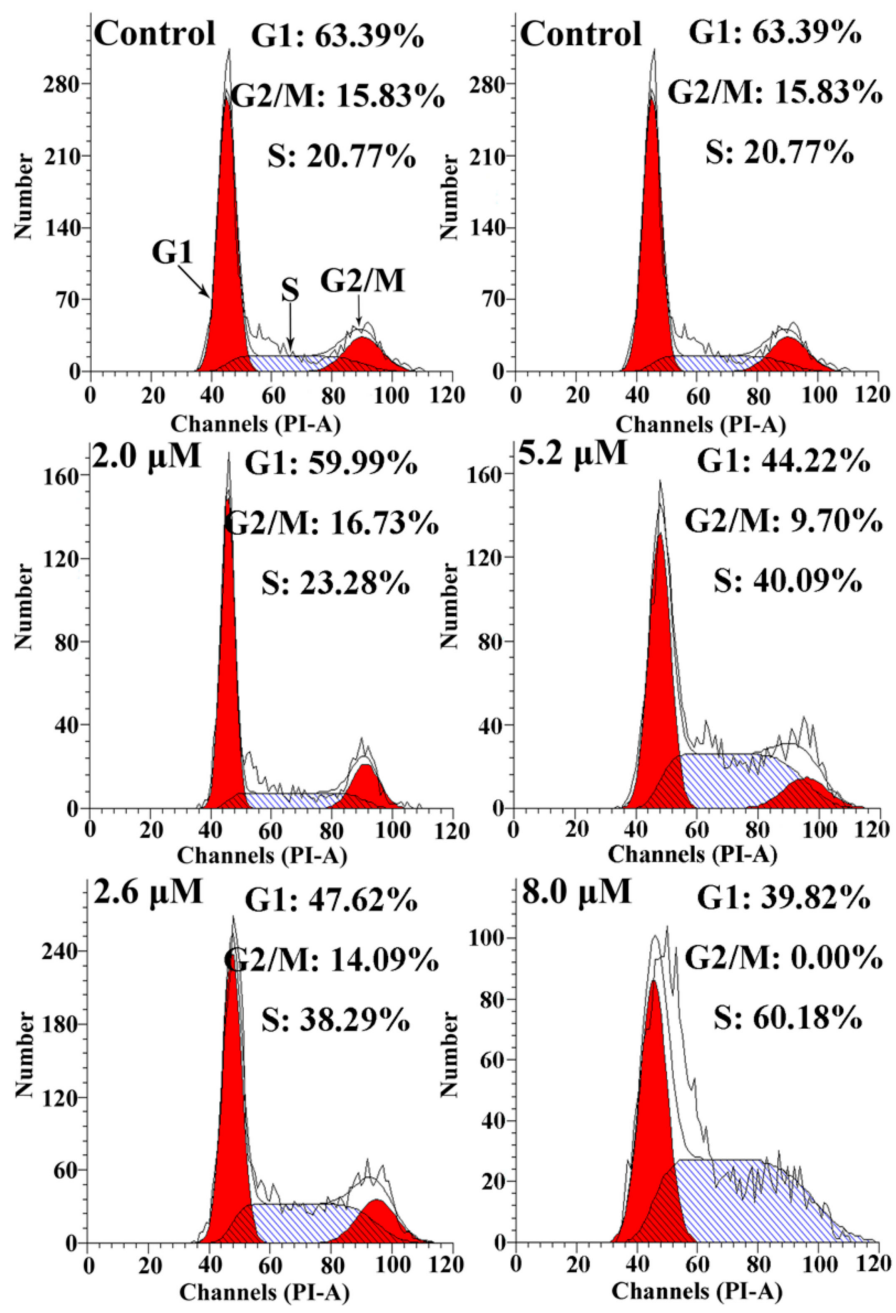
The cell cycle analysis by flow cytometry of MGC80-3 cells treated with complex **1** (2.0, 2.6, 5.2, and 8.0 µM) for 24 h.

The IC_50_ value of complex **1** towards the MGC80-3 cells was in the low micromolar range. To determine the cell cycle phase of growth arrest by complex **1**, the DNA content of cells was estimated by flow cytometry after the cells were stained with propidiumiodide (PI). As shown in Figure 3, complex **1** caused a dose-dependent accumulation of MGC80-3 cells in the S phase, whereas most of the control cells were in the G1 and G2/M phase of the cell cycle. Additionally, the cell population of S phase increased from 20.77% in the control to 60.18% in the MGC80-3 cells treated with 8 μM of complex **1** for 24 h. After incubating the cells with complex **1** (8 μM) for 24 h, the cell population of the G2/M phase was decreased to 0.00%. These results indicated that the MGC80-3 cells were mainly blocked in the S phase.

**Figure 4:**
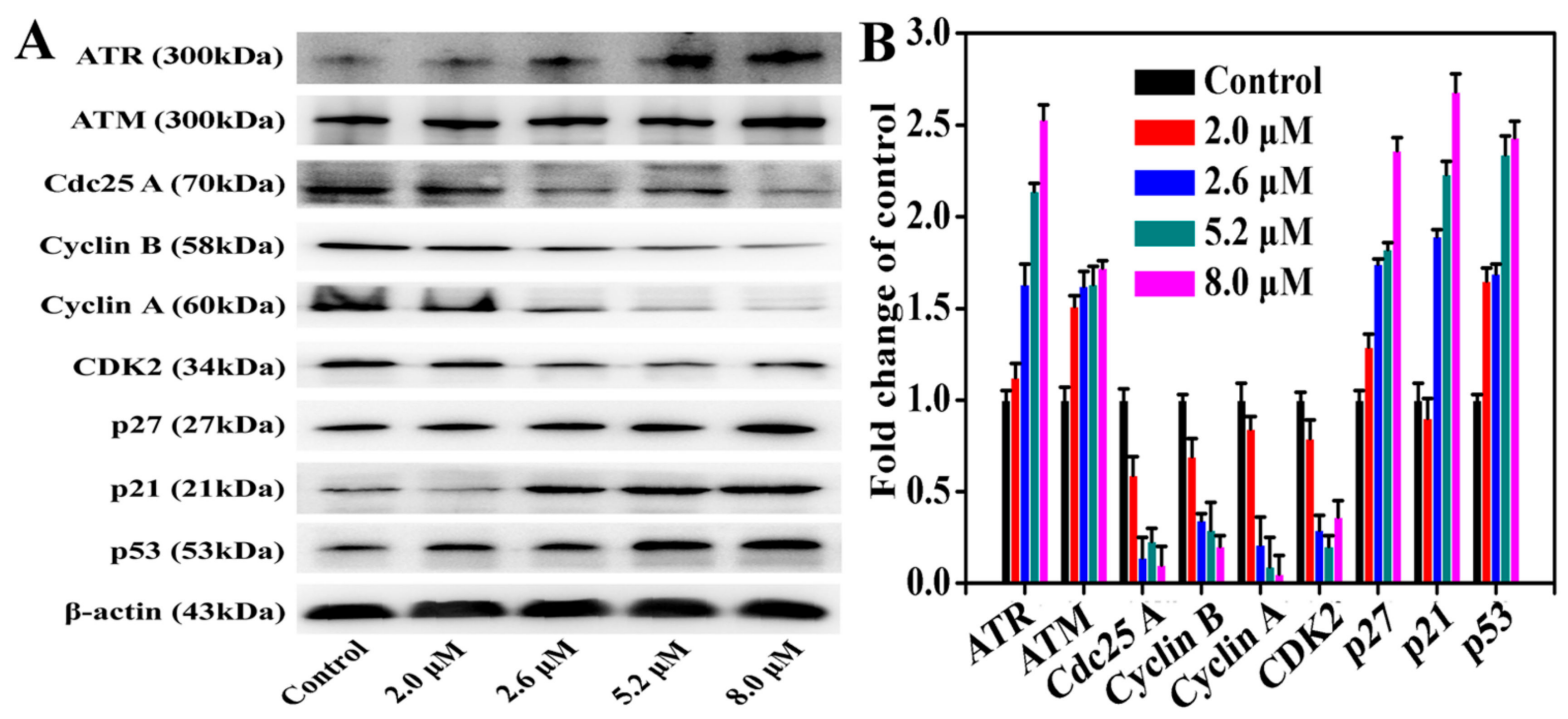
The expressions of ATR, ATM, Cdc25 A, Cyclin B, Cyclin A, CDK2, p27, p21, and p53 protein in MGC80-3 cells after treated with complex **1** (2.0, 2.6, 5.2, and 8.0 µM) for 24 h were analyzed by Western blot. (**A**) The same blots were stripped and re-probed with a β-actin antibody to show equal protein loading; (**B**) The whole-cell extracts were prepared and analyzed by Western blot analysis using antibodies against cell cycle protein regulator proteins. The same blots were stripped and re-probed with the β-actin antibody to show equal protein loading. Western blotting bands from three independent measurements were quantified with Image J in (**A**).

The protein expression levels of ATR, ATM, Cdc25 A, Cyclin B, Cyclin A, CDK2, p27, p21, and p53 protein in MGC80-3 cells after treated with complex **1** (2.0, 2.6, 5.2, and 8.0 µM) for 24 h were determined by Western blot and the results are shown in Figure 4, which demonstrated that complex **1** caused a dose-dependent inhibition on the protein expression levels of Cdc25 A, Cyclin B, Cyclin A, and CDK2, and decreased levels of p27, p21, and p53 proteins.

### 2.6. Apoptosis Assay

**Figure 5:**
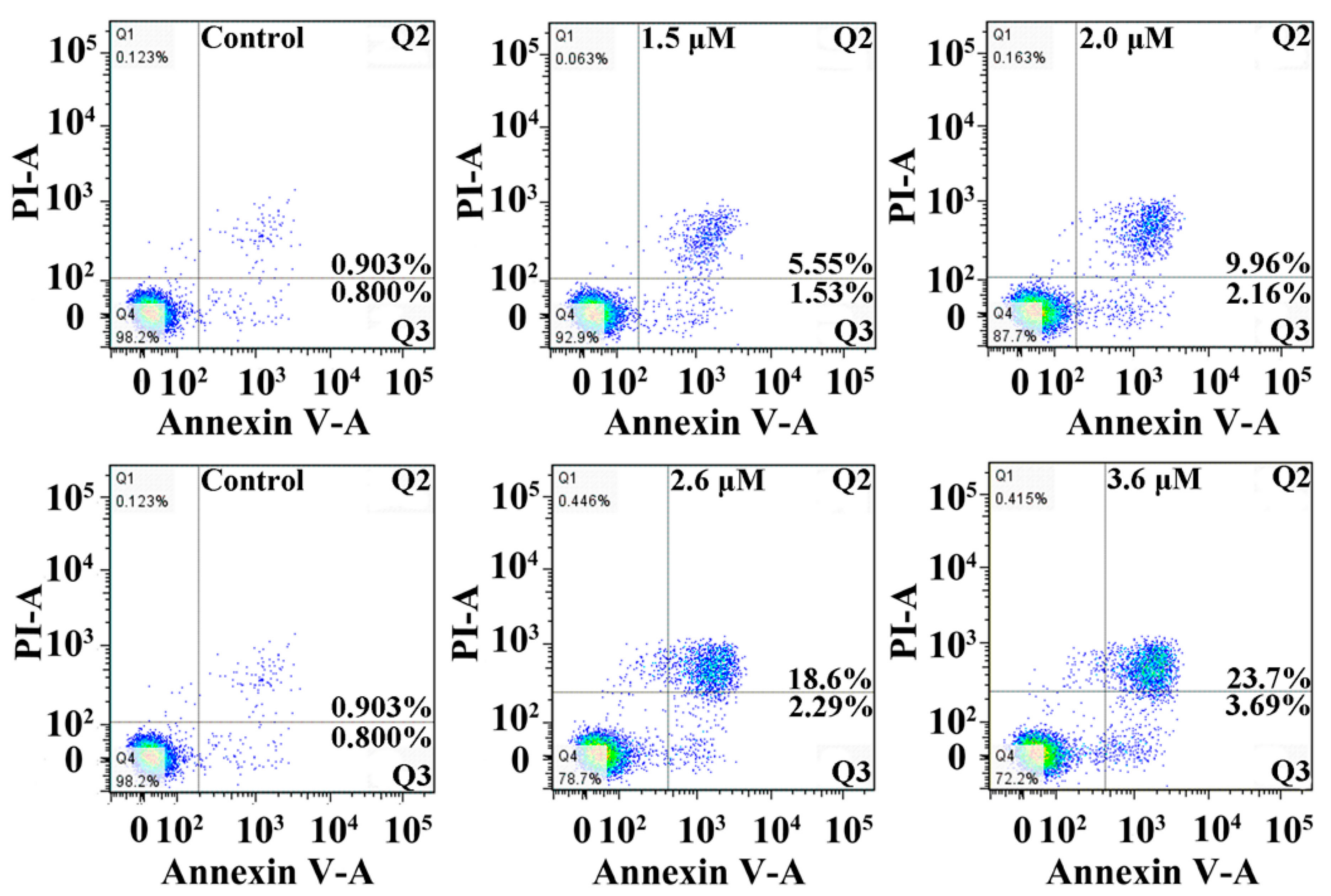
The Annexin V/propidium iodide assay and flow cytometry assay of MGC80-3 cells treated with Cu(II) complex **1** (1.5, 2.0, 2.6 and 3.6 µM).

Apoptosis assay can provide important information for the preliminary investigation of the mode of action [55,56,57]. To determine whether the death of MGC80-3 cells induced by complex **1** resulted from apoptosis or necrosis, common biochemical markers of apoptosis were monitored, including mitochondrial membrane depolarization, chromatin condensation, and phosphatidylserine exposure. The cells subjected to annexin V-FITC and PI staining were classified as necrotic cells (Q1; annexin V−/PI+), early apoptotic cells (Q2; annexin V+/PI−), late apoptotic cells (Q3; annexin V+/PI+), and intact cells (Q4; annexin V−/PI−). The assay showed (Figure 5) that complex **1** (1.5, 2.0, 2.6, and 3.6 µM) induced the apoptotic death of MGC80-3 cells as measured by annexin V staining and flow cytometry. After treatment with complex **1** for 24 h, the populations of apoptotic cells (Q2+Q3) changed from 7.08% to 27.39% with the increase of complex **1** concentration, but the population of apoptotic cells (Q2+Q3) of control was only 1.70%. The significantly increased percentages of apoptotic cells confirmed that complex **1** effectively induced MGC80-3 cell apoptosis in a dose-dependent manner, which was consistent with the results of the MTT assay.

### 2.7. Loss of Mitochondrial Membrane Potential in MGC80-3 Cells

**Figure 6:**
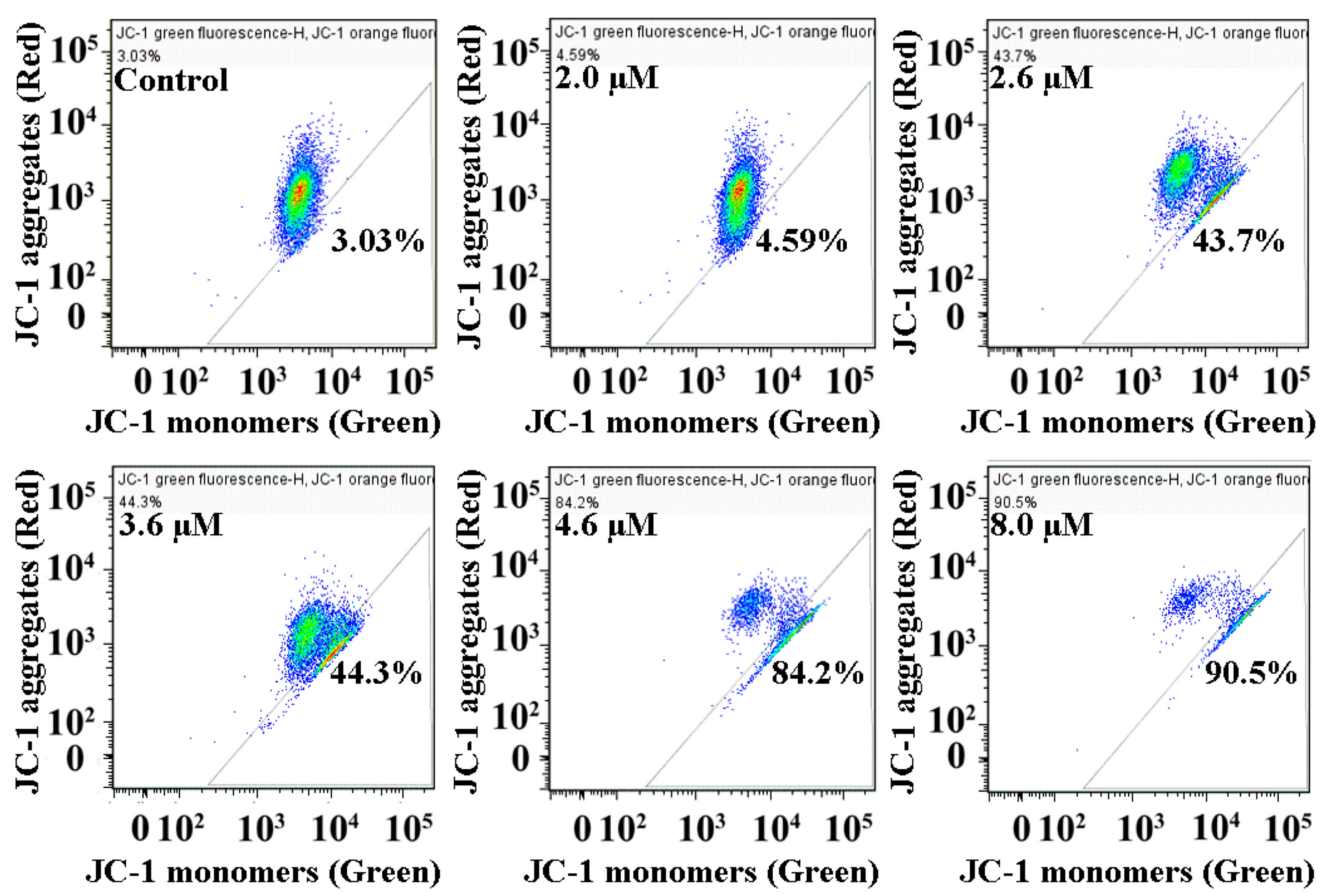
The collapse of mitochondrial membrane potential in MGC80-3 cells treated with Cu(II) complex **1** for 24 h, as determined by JC-1 staining.

**Figure 7:**
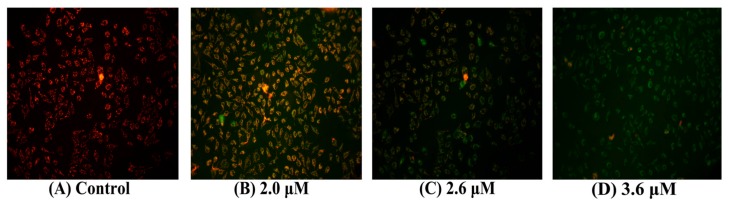
The loss of Δψ in MGC80-3 cells treated with complex **1** (2.0, 2.6 and 3.6 μM) for 24 h, and the cells were examined under a fluorescence microscope (Nikon Te2000, 200×) after being stained with JC-1.

Growing evidence has shown that mitochondria play a key role in the progression of apoptosis, and the loss of mitochondrial membrane potential (Δψ) is involved in apoptotic cell death due to the cytotoxicity of the antitumor compounds [58,59,60]. The changes in Δψ induced by complex **1** are shown in Figure 6 and Figure 7. JC-1 staining was used as a fluorescent probe [58]. After the MGC80-3 cells were treated with complex **1** for 24 h, the Δψ decreased significantly with the increase of dose (from 2.0 to 8.0 μM) of complex **1**, suggesting that the induction of apoptosis by complex **1** was associated with the intrinsic (mitochondrial) pathway.

### 2.8. Intracellular Ca^2+^

**Figure 8:**
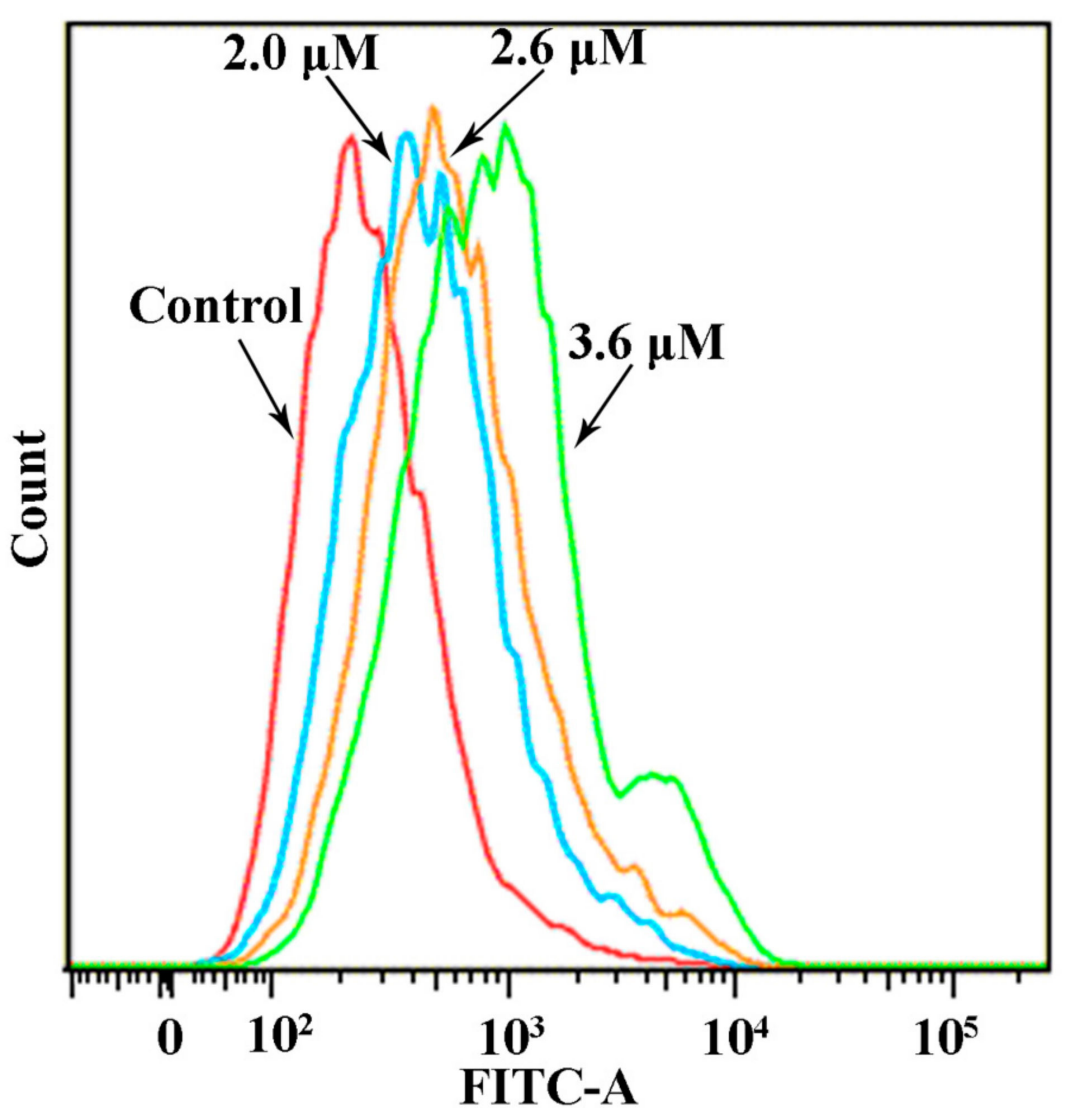
The effect of complex **1** (2.0, 2.6, and 3.6 μM) on the intracellular free Ca^2+^ level in MGC80-3 cells for 24 h.

The mitochondrial membrane potential Δψ can alter the intracellular Ca^2+^ level, which has been recognized as a factor in cell death, apoptosis, and injury mediated by various pathways [61,62]. We examined the effects of complex **1** on the mobilization of intracellular Ca^2+^ in MGC80-3 cells. As shown in Figure 8, the level of intracellular free Ca^2+^ in MGC80-3 cells was lower than that of the control group, but it increased steadily in a dose-dependent manner (2.0, 2.3, and 3.6 μM of complex **1**). Therefore, the changes of the intracellular Ca^2+^ level could be involved in the induction of apoptosis by complex **1** in MGC80-3 cells.

### 2.9. Reactive Oxygen Species (ROS) Level

**Figure 9:**
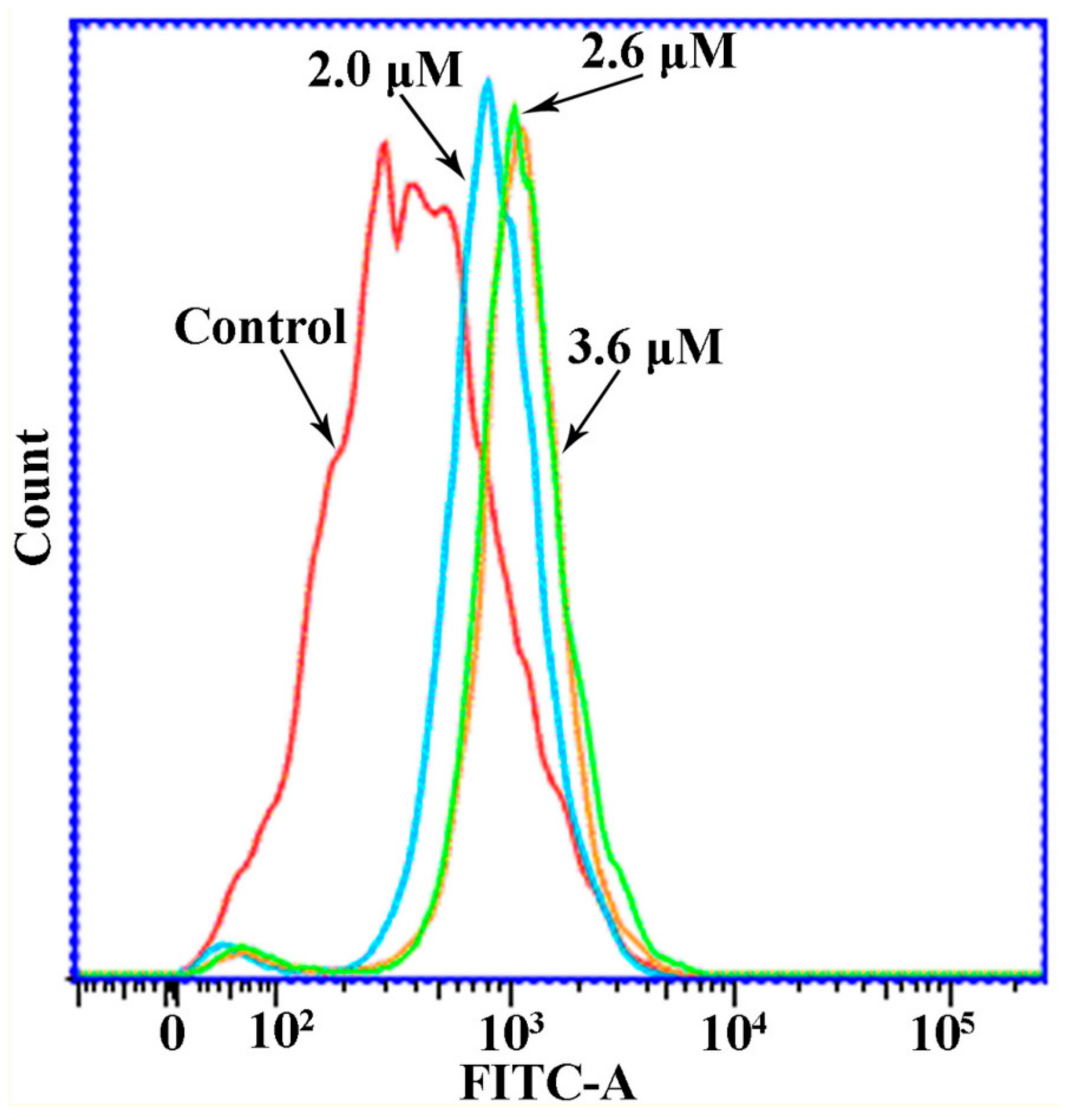
The ROS generation assay by flow cytometry analysis of MGC80-3 cells treated with complex **1** (2.0, 2.6, and 3.6μM). Results are expressed as relative fluorescent intensities (from left to right).

**Figure 10:**
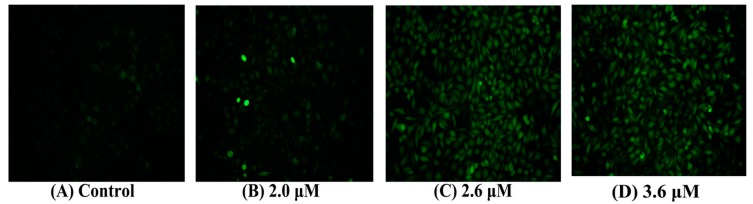
The ROS generation assay of MGC80-3 cells treated with complex **1** (2.0, 2.6, and 3.6 μM), and the cells were examined under a fluorescence microscope (Nikon Te2000, 200×).

The dysregulation of ROS generation could dramatically affect cancer cell structure and result in cell damage, and consequently cell death and apoptosis [63,64]. To determine whether ROS generation is involved in the apoptosis or death of MGC80-3 cells induced by complex **1**, the ROS level was measured by a fluorescent marker after the MGC80-3 cells were treated with complex **1** (2.0, 2.6, and 3.6 μM) for 24 h. As shown in Figure 9 and Figure 10, the levels of ROS in MGC80-3 cells were higher than that in the control after treatment, and the levels of ROS increased in a dose-dependent manner (from 2.0 to 3.6 μM of complex **1**). The results confirmed that complex **1** stimulated ROS-induced apoptosis in MGC80-3 cells.

### 2.10. Western Blot Assay

**Figure 11:**
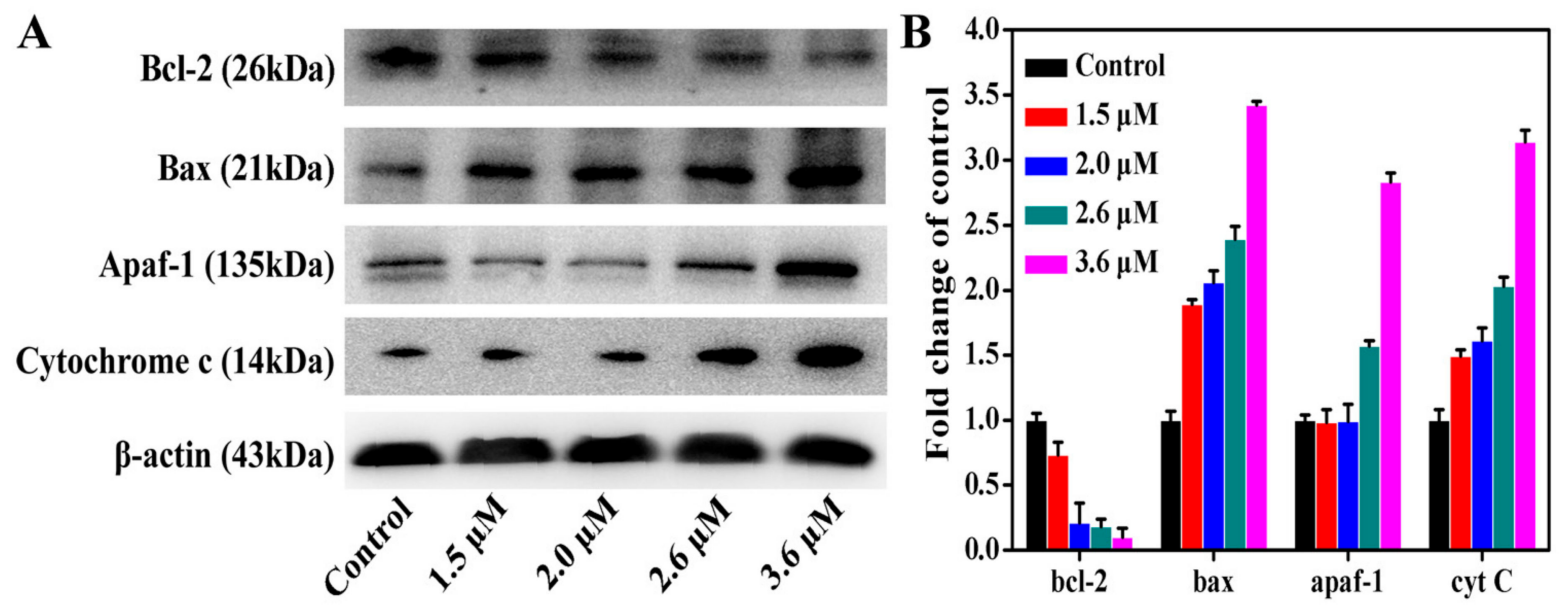
The western blot assay of apoptosis-related protein levels in Hep-G2 cells treated with complex **1** (1.5, 2.0, 2.6, and 3.6 μM) for 24 h. (**A**) Western blot was used to determine the expression levels of bax, cytochrome c, apaf-1, and bcl-2 in MGC80-3 cells treated with complex **1** (1.5, 2.0, 2.6, and 3.6 μM) for 24 h; (**B**) Densitometric analysis of apoptotic-related proteins normalized to β-actin. The relative expression of each protein is represented by the density of the protein band/density of β-actin band.

To further investigate the mechanism of action of complex **1**, the cytochrome C (Cyt C), bcl-2, bax, and apaf-1 proteins in the mitochondria-related apoptotic pathway were assayed by Western blot [65]. Figure 11 shows that the levels of bax, Cyt C, and apaf-1 proteins increased significantly and the level of bcl-2 protein decreased significantly in the MGC80-3 cells after treatment with complex **1** (1.5, 2.0, 2.6, and 3.6 μM) for 24 h. Additionally, the levels of bax, Cyt C, and apaf-1 proteins increased in a dose-dependent manner. These results further demonstrated that complex **1** may be involved in mitochondria-related apoptosis [65].

### 2.11. Assessment of Caspase-3/9/8 Activation

**Figure 12:**
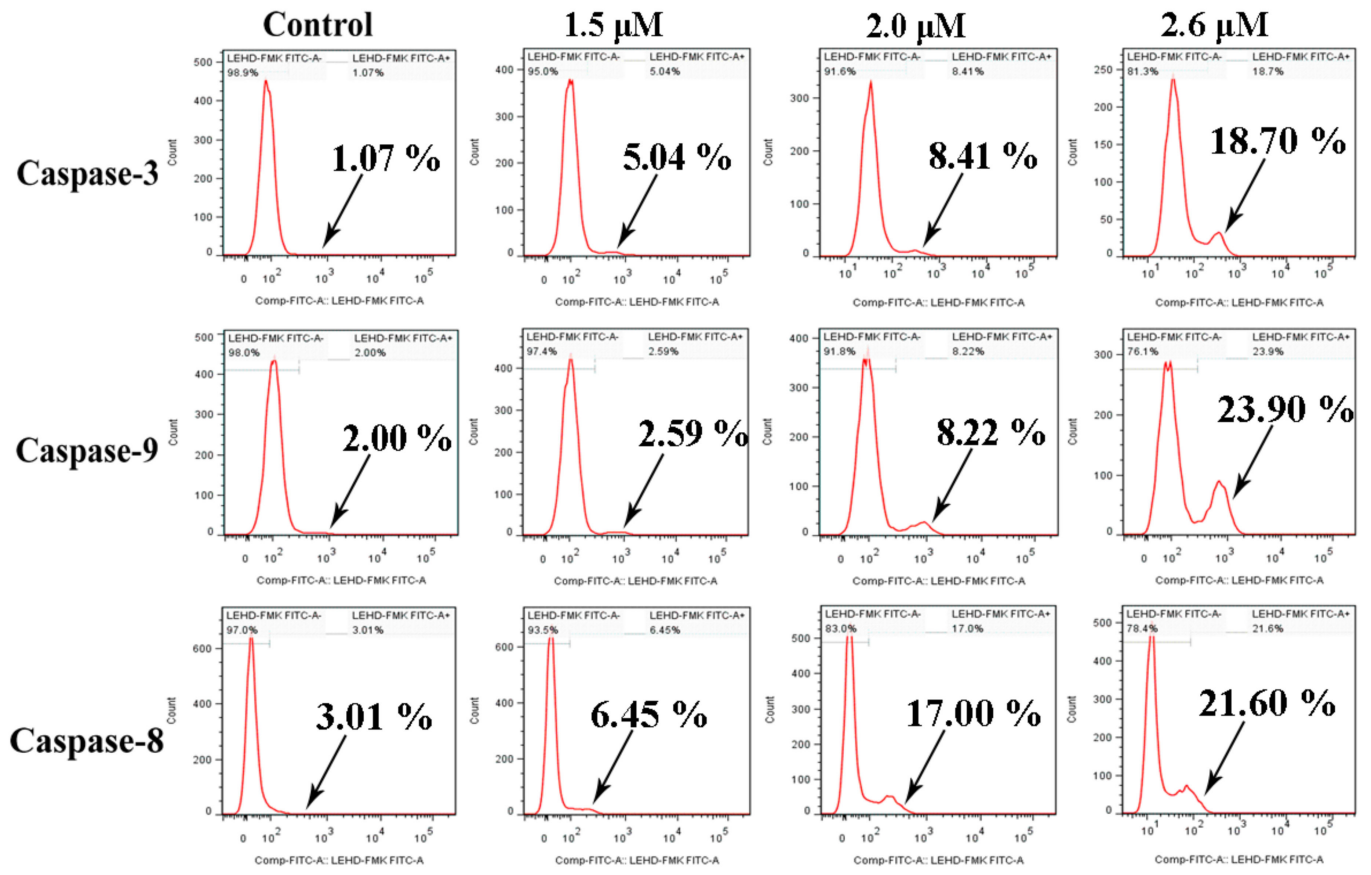
The activation of caspase-3, caspase-8, and caspase-9 in MGC80-3 cells treated with complex **1** (1.5, 2.0, and 2.6 μM) for 24 h.

To determine whether caspase-3/9 were involved in the induced apoptotic cell death, MGC80-3 cells were analyzed by flow cytometry after treatment with complex **1** (1.5, 2.0, and 2.6 μM) for 24 h. The results showing peaks of activated caspase-3 (FITC-DEVD-FMK probes), activated caspase-8 (FITC-IETD-FMK probes), and activated caspase-9 (FITC-LEHD-FMK probes) for the treated cells are shown in Figure 12. It is notable that the proportion of cells with activated caspase-3, caspase-9, and caspase-8 increased from 5.04% to 18.70%, 2.59% to 23.9%, and 6.45% to 21.60%, respectively. Therefore, complex **1** could induce cell apoptosis by triggering the caspase-3/9/8 activity in MGC80-3 cells [66,67,68,69].

Taken together, complex **1** induced apoptosis in MGC80-3 cells likely by disrupting mitochondrial function, which led to a significantly decreased level of bcl-2 protein and loss of Δψ, as well as a significant increase in the levels of ROS, intracellular Ca^2+^, Cyt C protein, apaf-1 protein, activated caspase-3, and activated caspase-9 in MGC80-3 cells.

## 3. Materials and Methods

### 3.1. Materials

All chemical reagents, including chloride salts and solvents, were of analytical grade. All materials were used as received without further purification unless specifically noted. All the synthetic complexes were dissolved in dimethyl sulfoxide (DMSO) for the preparation of stock solution at a concentration of 2.0 mM.

### 3.2. Instrumentation

Elemental analyses (C, H, N) were carried out on a Perkin Elmer Series II CHNS/O 2400 elemental analyzer. NMR spectra were recorded on a Bruker AV-500 NMR spectrometer. Fluorescence measurements were performed on a Shimadzu RF-5301/PC spectrofluorophotometer. The region between 200 and 400 nm was scanned for each sample. UV-Vis spectra were recorded on a TU-1901 ultraviolet spectrophotometer.

### 3.3. Synthesis

#### 3.3.1. Synthesis of L

The 2-((2-(pyridin-2-yl)hydrazono)methyl)quinolin-8-ol (L) was obtained from the condensation reaction of 8-hydroxyquinoline-2-carbaldehyde with 2-hydrazinylpyridine in good yield (89.0%) [44], as shown in Scheme 2. m.p. 238 °C; ^1^H-NMR (500 MHz, DMSO-*d*_6_) δ 11.46 (s, 1H, OH), 9.72 (s, 1H, NH), 8.27 (d, *J* = 9.0 Hz, 2H), 8.18 (d, *J* = 4.0 Hz, 1H), 8.14 (d, *J* = 8.6 Hz, 1H), 7.74–7.68 (m, 1H), 7.42–7.36 (m, 3H), 7.09 (dd, *J* = 7.2, 1.5 Hz, 1H), 6.85 (dd, *J* = 6.9, 5.1 Hz, 1H); ^13^C-NMR (125 MHz, DMSO-*d*_6_) δ 157.10, 153.61, 153.16, 148.32, 139.65, 138.60, 138.52, 136.57, 128.71, 127.87, 118.22, 117.91, 116.27, 112.41, 107.19; HRMS(EI): Calcd for C_15_H_13_N_4_O [L + H]^+^, *m*/*z* 265.1089, found *m*/*z* 265.1086. IR (cm^−1^): ν_NH_ = 3049 cm^−1^; ν_C=N_ = 1580 cm^−1^. (The ^1^H-NMR, ^13^C-NMR and MS were shown in Figures S6–S8)

#### 3.3.2. Synthesis of Complex **1**

The mixture of ligand L (0.26 g, 1.0 mmol) and CuCl_2_·2H_2_O (0.17 g, 1.0 mmol) in 20 mL methanol was maintained at reflux (70 °C) for 6 h to afford complex **1** as black crystals in 70% yield. The black crystals of complex **1** suitable for X-ray diffraction analysis were subsequently harvested. ESI-MS *m*/*z*: 427.0361[Cu(L)Cl + H + 2MeOH]^+^. Anal. Calcd for C_30_H_24_Cl_4_Cu_2_N_8_O_2_: C, 45.18; H, 3.03; N, 14.05; O, 4.01; Found: C, 45.16; H, 3.02; N, 15.07. IR (cm^−1^): ν_NH_ = 3101 cm^−1^; ν_C=N_ = 1636 cm^−1^. (The MS was shown in Figure S9)

#### 3.3.3. Synthesis of Complex **2**

By means of the similar procedure, complex **2** was obtained from NiCl_2_·6H_2_O as black crystals in 75% yield. The black crystals of complex **2** suitable for X-ray diffraction analysis were subsequently harvested. ESI-MS *m*/*z*: 321.0281 [Ni(L) + H]^+^. Anal. calcd for C_16_H_14_Cl_4_N_4_NiO: C, 40.13; H, 2.95; N, 11.70; Found: C, 40.14; H, 2.94; N, 11.69. IR (cm^−1^): ν_NH_ = 3060 cm^−1^; ν_C=N_ = 1615 cm^−1^. (The MS was shown in Figure S10)

### 3.4. X-ray Crystallography

Complexes **1** (0.31 × 0.22 × 0.10 mm) and **2** (0.34 × 0.18 × 0.17 mm) were measured on an Agilent SuperNova CCD area detector (Rigaku Corporation, Tokyo, Japan) equipped with a graphite-monochromatic Mo-Kα radiation source (λ = 0.71073 Ǻ) at room temperature 293(2) K. All non-hydrogen atoms’ positions and anisotropic thermal parameters were refined on F2 by full-matrix least-squares techniques with the SHELX-97 program package [70]. The hydrogen atoms were added theoretically, riding on the concerned atoms. The semi-empirical methods from equivalents were used to correct absorption. The crystallographic data and refinement details of the structures are summarized in Tables S1–S3 (Supporting Information).

### 3.5. In Vitro Cytotoxicity

Seven tumor cells Hep-G2, SK-OV-3, MGC80-3, HeLa, T-24, BEL7402, and NCI-H460 and one normal liver cell HL-7702 were obtained from the Shanghai Cell Bank in the Chinese Academy of Sciences. Cells were grown in triplicate in 96-well plates (Gibco, Carlsbad, CA, USA) and incubated at 37 °C for 48 h in a humidified atmosphere containing 5% CO_2_ and 95% air. To investigate the potential activity of L and complexes **1** and **2**, cisplatin was employed as a reference metallodrug. Cytotoxicity assays were carried out in 96-well flat-bottomed microtite plates that were supplemented with culture medium and cells. Ligand L, complexes **1** and **2**, and cisplatin were dissolved in the culture medium at various concentrations (1.25, 2.5, 5.0, 10.0, and 20.0 μM) with 1% DMSO and the resulting solutions were subsequently added to a set of wells. The control wells contained supplemented medium with 1% DMSO. The microtitre plates were then incubated at 37 °C under a humidified atmosphere containing 5% CO_2_ and 95% air for 2 days. Cytotoxicity screening was conducted through 3-(4,5-dimethylthiazol-2-yl)-2,5-diphenyltetrazolium bromide (MTT) assay. After each incubation period, the MTT solution (10 mL, 5 mg·mL^−1^) was added into each well and the cultures were incubated at 37 °C in a humidified atmosphere containing 5% CO_2_ and 95% air for a further 48 h. After the removal of the supernatant, DMSO (150 mL) was added to dissolve the formazan crystals.

The absorbance at 490 and 630 nm was read on a plate reader. Relative to the negative control, cytotoxicity was estimated based on the percentage cell survival in a dose-dependent manner. The final IC_50_ values were calculated by the Bliss method (*n* = 5). All tests were repeated in at least three independent experiments.

### 3.6. Cell Cycle Arrest

The MGC80-3 cells were maintained in Dulbecco’s modified Eagle’s medium with 10.0% fetal calf serum under 5% CO_2_ at 37 °C. The cells were harvested by trypsinization, rinsed with PBS, and centrifuged at 3000× *g* for 10 min. The pellet (105–106 cells) was suspended in PBS (1.0 mL) and kept on ice for 5 min. The cell suspension was then fixed by the dropwise addition of 9 mL precooled (4 °C) 100% ethanol with vigorous shaking, and the fixed samples were kept at 4 °C until use. For staining, the cells were centrifuged, resuspended in PBS, digested with 150 mL RNase A (250 μg·mL^−1^), treated with 150 mL PI (0.15 mM), and then incubated for 30 min at 4 °C. PI-positive cells were counted with a fluorescence-activated cell sorter (FACS). The population of cells in each cell cycle was determined by the Cell Modi FIT software (Becton Dickinson, version 1.0, San Jose, CA, USA).

### 3.7. Other Experimental Methods

The supporting information provides the detailed procedures of other experimental methods, including the measurement of mitochondrial membrane potential (by JC-1 staining), ROS generation, intracellular free Ca^2+^, Western blot, and caspase-3/9 activity. The procedures were similar to those given in the previous work of Chen et al. [71].

### 3.8. Statistics

Data processing included the Student’s *t*-test with *p* ≤ 0.05 taken as significance level, using SPSS 13.0 (IBM, Armonk, NY, USA).

## 4. Conclusions

Two transition metal complexes with 2-((2-(pyridin-2-yl) hydrazono) methyl)quinolin-8-ol (L), [Cu(L)Cl_2_]_2_ (**1**) and [Ni(L)Cl_2_]·CH_2_Cl_2_ (**2**), were synthesized and fully characterized. In vitro antitumor screening revealed that complex **1** exhibited higher inhibitory activities than cisplatin against SK-OV-3, MGC80-3, and HeLa cells. In addition, complex **1** caused MGC80-3 cell arrest in the S phase, which led to the significant down-regulation of the related proteins. Complex **1** can down-regulate the expression of the bcl-2 protein and upregulate the levels of the bax, Cyt C, and apaf-1 proteins in MGC80-3 cells. We found that complex **1** induced MGC80-3 cell apoptosis via a mitochondrial dysfunction pathway, which was mediated by Δψ, ROS, and intracellular Ca^2+^. Moreover, complex **1** could induce cell apoptosis by triggering the caspase-3/9/8 activity in MGC80-3 cells. Therefore, complex **1** is a potent anticancer drug candidate.

## Figures and Tables

**Table 1:** The IC_50_^a^ (μM) values of L, complexes **1** and **2** to the selected tumor cells for 48 h.

Compounds	Hep-G2	SK-OV-3	MGC80-3	HeLa	T-24	BEL-7402	NCI-H460	HL-7702
L	58.40 ± 0.69	42.94 ± 2.64	40.93 ± 0.94	58.73 ± 1.29	85.93 ± 15.11	47.85 ± 0.37	36.93 ± 3.93	48.63 ± 0.34
**1**	4.51 ± 0.38	3.69 ± 0.16	2.60 ± 0.17	3.62 ± 0.12	4.41 ± 0.06	5.92 ± 0.01	5.01 ± 0.16	12.78 ± 0.55
**2**	>100	39.77 ± 2.15	38.99 ± 2.42	27.13 ± 6.51	18.97 ± 3.47	51.68 ± 0.66	36.31 ± 3.75	31.73 ± 2.11
Cisplatin ^b^	9.55 ± 0.46	16.32 ± 1.37	12.37 ± 1.53	9.45 ± 2.05	28.15 ± 1.67	19.4 ± 0.58	9.59 ± 0.48	15.87 ± 0.36

^a^ IC_50_ values are presented as the mean ± SD (standard error of the mean) from five separate experiments. ^b^ Cisplatin was dissolved at a concentration of 1 mM in 0.154 M NaCl.

## Supplementary Materials

Can be found at http://www.mdpi.com/1422-0067/19/7/1874/s1, Full cif depositions (excluding structure factors) lodged with the Cambridge Crystallographic Data Centre (CCDC 1848527 (for complexes **1**), 1848516 (for complexes **2**)) contain the supplementary crystallographic data for this paper. These data can be obtained free of charge from The Cambridge Crystallographic Data Centre via www.ccdc.cam.ac.uk/data_request/cif.
