# Supplementary material for: Syntheses, Crystal Structures, and Antitumor Activities of Copper(II) and Nickel(II) Complexes with 2-((2-(Pyridin-2-yl)hydrazono)methyl)quinolin-8-ol
Source: Int J Mol Sci. 2018 Jun 26;19(7):1874. doi: 10.3390/ijms19071874 (PMC6073241; doi:10.3390/ijms19071874)
Supplement: Supplementary file 1 [file ijms-19-01874-s001.pdf]

## Supplementary data

# Syntheses, crystal structures and antitumor activities of copper(II) and nickel(II) complexes with 2-((2-(pyridin-2-yl) hydrazono) methyl) quinolin-8-ol

Qi-Yuan Yang†, Qian-Qian Cao†, Qi-Pin Qin, Cai-Xing Deng, Hong Liang*, Zhen-Feng Chen*

**Table S1.** Crystallographic data and refinements of complexes **1-2**.

| | | |
|---|---|---|
| Formula | $C_{30}H_{28}Cl_4Cu_2N_8O_4$ | $C_{15.5}H_{13}Cl_3N_4NiO$ |
| Mr | 833.5 | 436.36 |
| Crystal system | Triclinic | Monoclinic |
| Space group | P-1 | C2/c |
| a/Å | 7.2964(6) | 19.4265(15) |
| b/Å | 12.525(2) | 16.5159(11) |
| c/Å | 18.5544(13) | 10.8190(7)<br>90.00 |
| α/° | 98.673(10) | 100.521(7) |
| β/° | 90.269(7) | 90.00 |
| γ/° | 100.984(12) | 3412.9(4) |
| V/Å$^3$ | 1644.6(4) | 982.69(1) |
| T/K | 293(2) | 293(2) |
| Z | 2 | 8 |
| $D_c$/g.cm$^{-3}$ | 1.683 | 1.698 |
| θ/° | 6.66 to 50.7° | 6.86 to 52.74° |
| *F* (000) | 844.0 | 1768.0 |
| μ (Mo, Kα)(mm$^{-1}$) | 1.669 | 1.618 |
| Total no. reflns | 18393 | 17263 |
| No. indep. reflns | 6005 | 3492 |
| $R_{int}$ | 0.0714 | 0.0554 |
| R1 [I > 2σ (I)] | 0.1181 | 0.0507 |
| ωR2(all data) | 0.3269 | 0.1408 |
| Gof(F$^2$) | 1.090 | 1.054 |

**Table S2.** Selected bond lengths (Å) and angles (°) for complex **1**.

| Bond names | Bond length(Å) | Bond angle | Angle(°) |
|---|---|---|---|
| Cu1−N1 | 2.004(9) | N1−Cu1−N3 | 80.3(3) |
| Cu1−N3 | 2.049(10) | N1−Cu1−Cl2 | 172.8(3) |
| Cu1−Cl2 | 2.254(3) | N3−Cu1−Cl2 | 92.6(2) |
| Cu1−Cl1 | 2.267(4) | N1−Cu1−Cl1 | 93.8(3) |
| Cu1−Cl2a | 2.706(4) | N3−Cu1−Cl1 | 159.8(3) |
| Cl2−Cu1a | 2.706(4) | Cl2−Cu1−Cl1 | 92.81(13) |
| O1−C14 | 1.366(14) | N1−Cu1−Cl2a | 92.2(3) |
| N1−C5 | 1.333(14) | N3−Cu1−Cl2a | 95.3(3) |
| N1−C1 | 1.340(14) | Cl2−Cu1−Cl2a | 88.94(11) |
| N3−C6 | 1.300(15) | Cl1−Cu1−Cl2a | 104.27(17) |
| N3−N2 | 1.351(11) | Cu1−Cl2−Cu1a | 91.06(11) |
| C4−C3 | 1.354(15) | C5−N1−C1 | 118.0(9) |
| C4−C5 | 1.367(13) | C5−N1−Cu1 | 113.0(7) |
| N2−C5 | 1.339(14) | C1−N1−Cu1 | 128.9(7) |
| N4−C7 | 1.320(13) | C6−N3−N2 | 119.0(10) |
| N4−C15 | 1.359(14) | C6−N3−Cu1 | 131.2(7) |

**Table S3.** Selected bond lengths (Å) and angles (°) for complex **2**.

| Bond names | Bond length(Å) | Bond angle | Angle(°) |
|---|---|---|---|
| Ni1−Cl2 | 2.3266(13) | Cl3−Ni1−Cl2 | 155.72(5) |
| Ni1−Cl3 | 2.3083(13) | N1−Ni1−Cl2 | 96.18(10) |
| Ni1−N1 | 2.077(3) | N1−Ni1−Cl3 | 92.37(10) |
| Ni1−N | 1.985(3) | N1−Ni1−N10 | 155.58(3) |
| Ni1−N10 | 2.126(4) | N−Ni1−Cl2 | 98.02(11) |
| N1−C8 | 1.333(5) | N−Ni1−Cl3 | 105.93(1) |
| N1−C13 | 1.361(5) | N−Ni1−N1 | 78.85(14) |
| O−C12 | 1.355(5) | N−Ni1−N10 | 77.17(14) |
| Cl1A−C26 | 1.648(6) | N10−Ni1−Cl2 | 91.66(10) |
| C4−C13 | 1.421(6) | N10−Ni1-Cl3 | 89.81(10) |
| C4−C24 | 1.408(7) | C8−N1−Ni1 | 112.1(3) |
| C4−C | 1.404(7) | C8−N1−C13 | 119.1(4) |
| N−C6 | 1.287(5) | C13−N1−Ni1 | 128.6(3) |

**Table S4.** Inhibitory rates (%) of L, complexes **1** and **2** (20.0μM) towards seven selected

tumour cells and one normal liver cells for 48 h.

| | Hep-G2 | SK-OV-3 | MGC80-3 | HeLa | T-24 | BEL-7402 | NCI-H460 | HL-7702 |
|---|---|---|---|---|---|---|---|---|
| **L** | 24.51±0.72 | 30.04±7.63 | 28.80±1.10 | 14.25±0.47 | 27.85±5.12 | 30.08±0.62 | 31.81±1.41 | 12.94±1.08 |
| **1** | 90.37±0.28 | 83.57±1.18 | 87.89±0.56 | 76.51±2.29 | 86.15±0.52 | 95.00±0.40 | 79.80±0.85 | 79.55±1.98 |
| **2** | 25.24±3.95 | 32.56±2.63 | 34.17±1.31 | 47.77±1.80 | 50.11±5.38 | 28.99±1.17 | 37.50±4.17 | 28.80±1.49 |

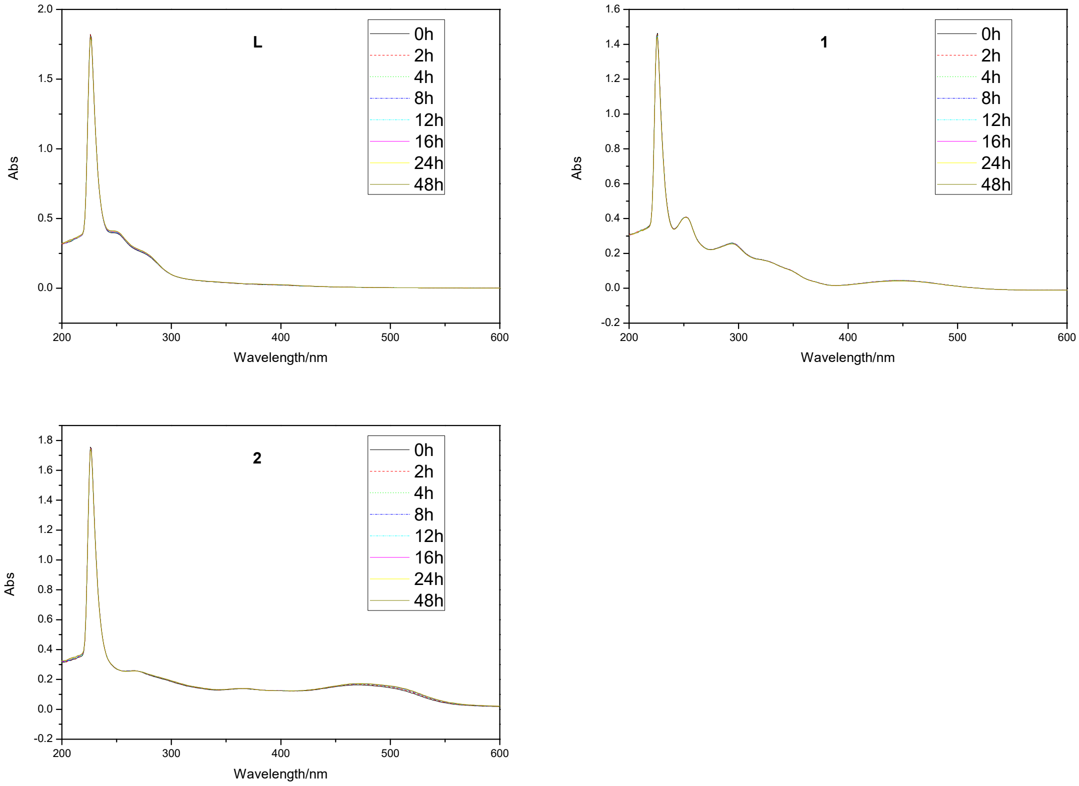


**Figure S1.** Time-dependent stability studies on L, complexes**1**and**2** in PBS monitored by UV-vis absorption spectra.

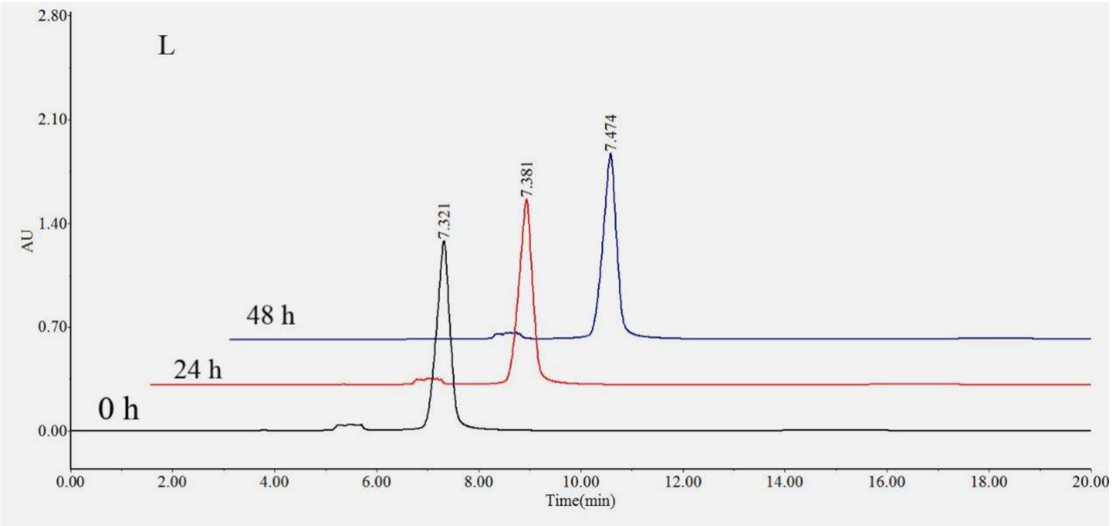

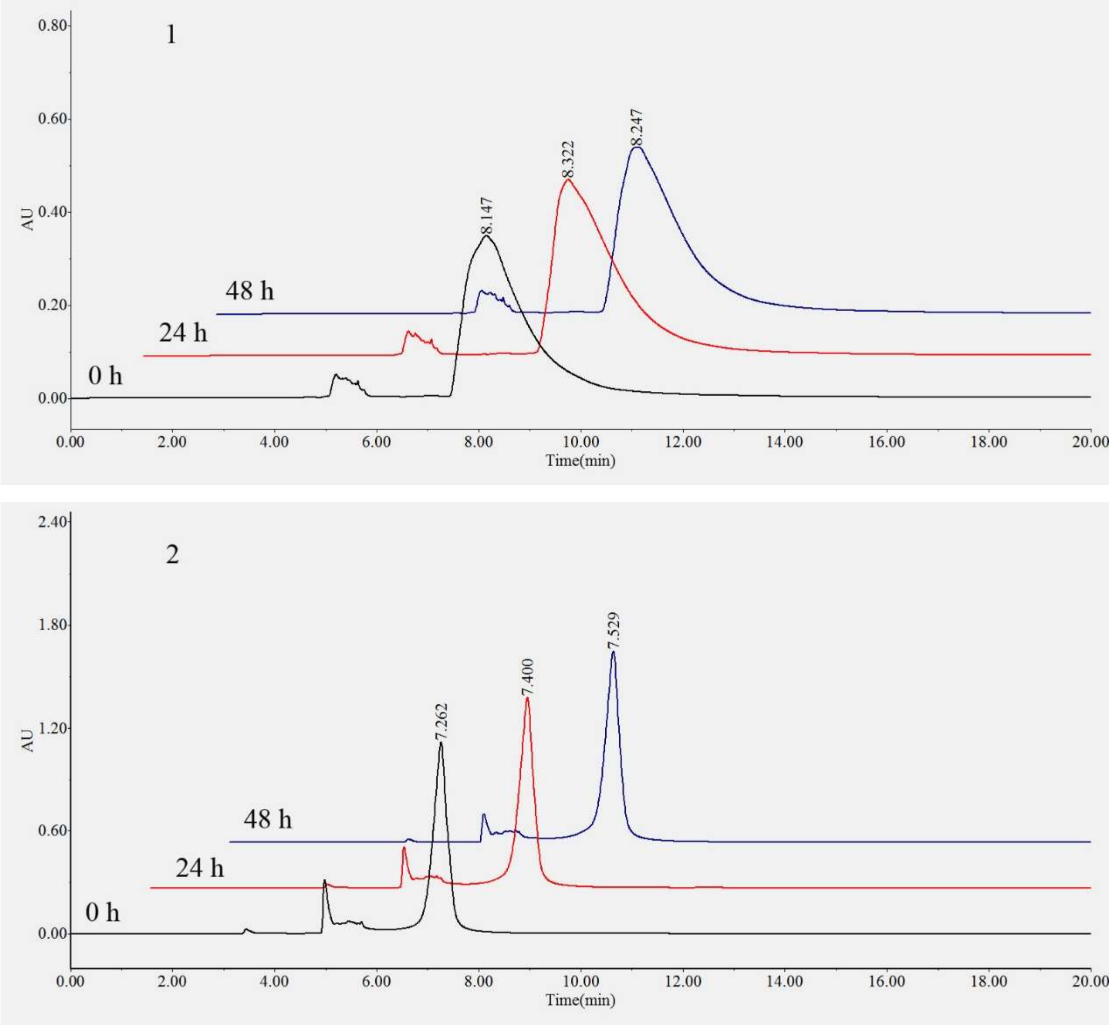


**Figure S2.** Time-dependent stability studies on L, complexes**1**and**2** in TBS monitored by HPLC.

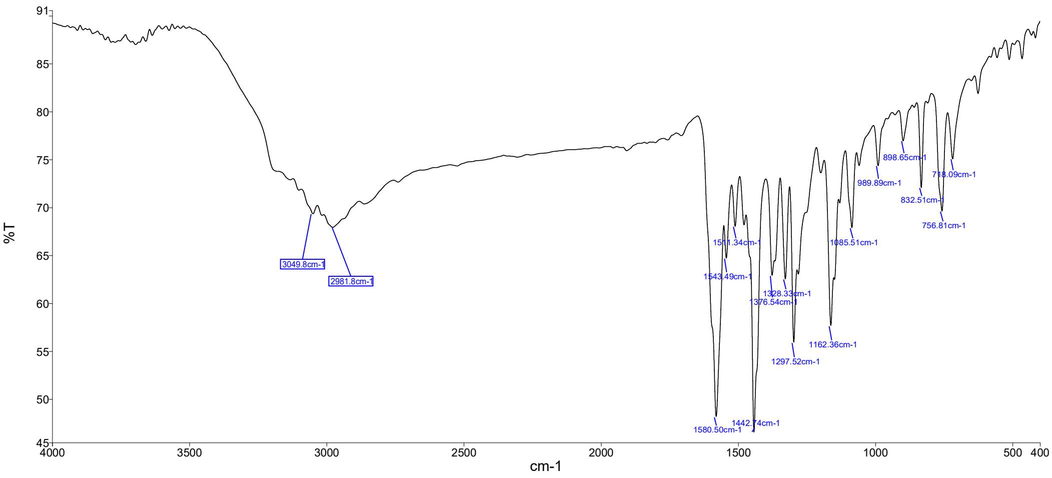


**Figure S3.** IR of ligand **L**.

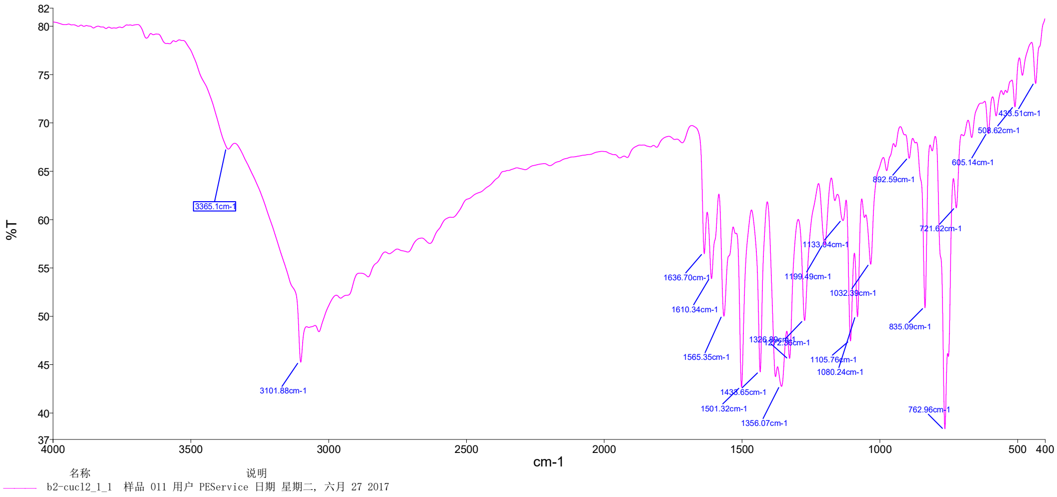


**Figure S4.** IR of complex **1**

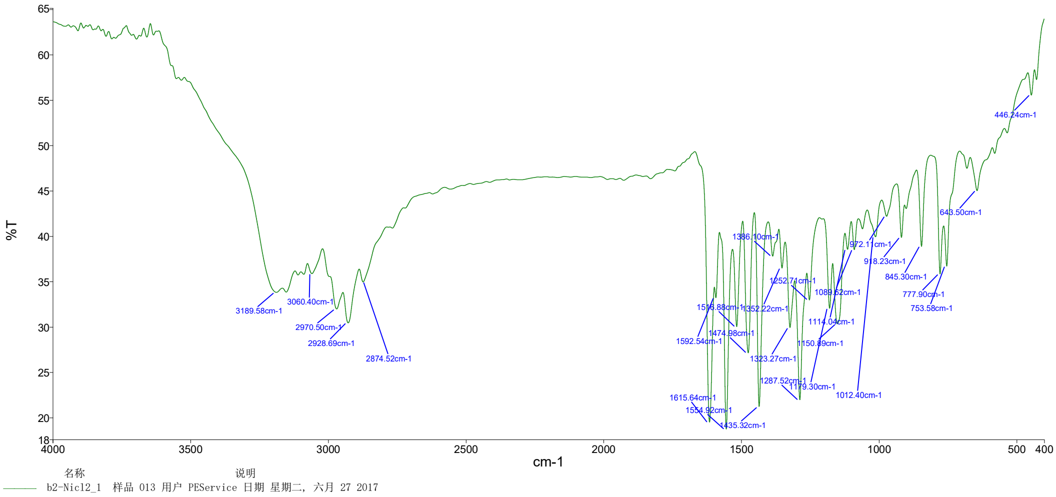


**Figure S5.** IR of complex **2**.

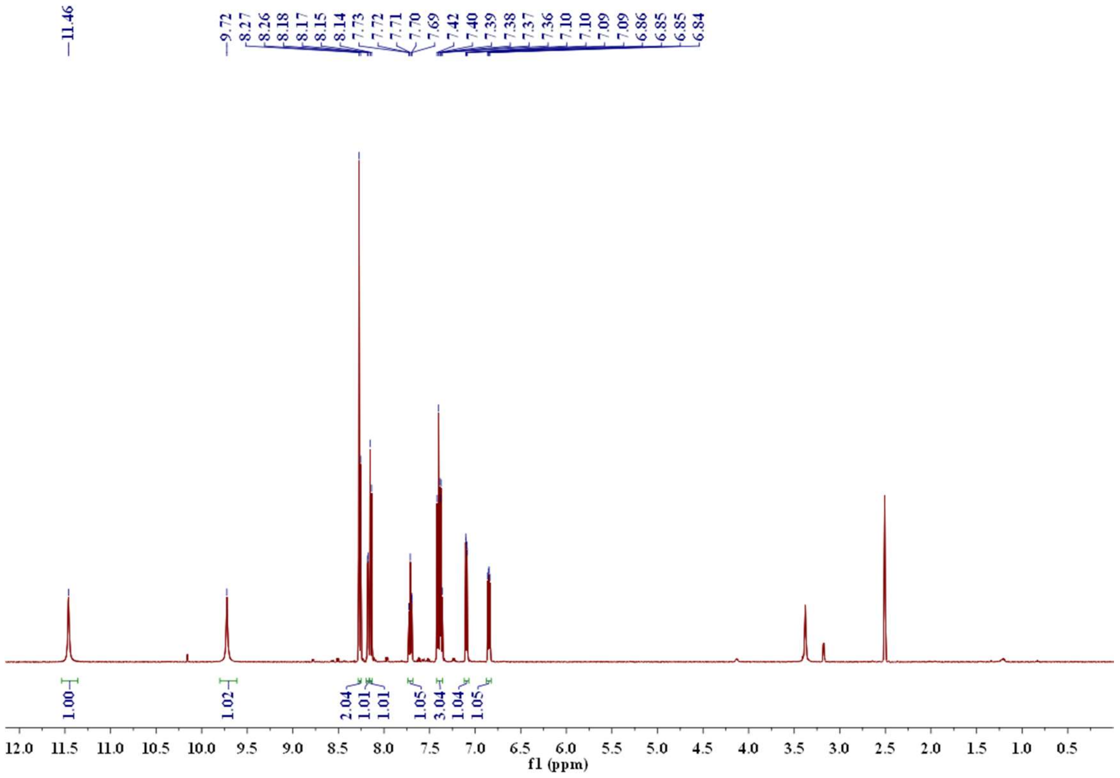


**Figure S6.** $^{1}H$ NMR of ligand **L**.

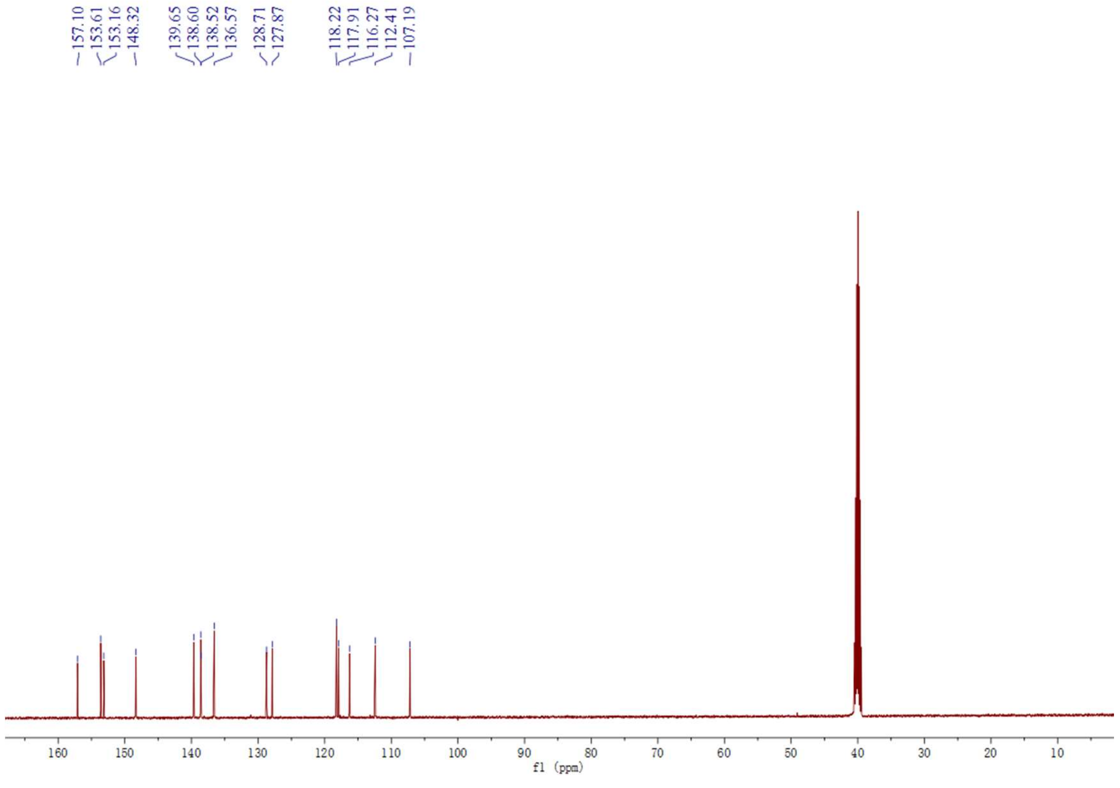


**Figure S7.** $^{13}C$ NMR of ligand **L**.

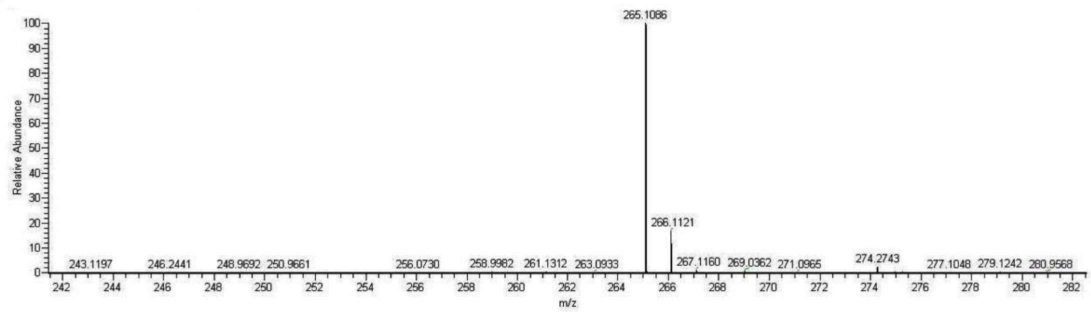


**Figure S8.** MS of ligand **L**.

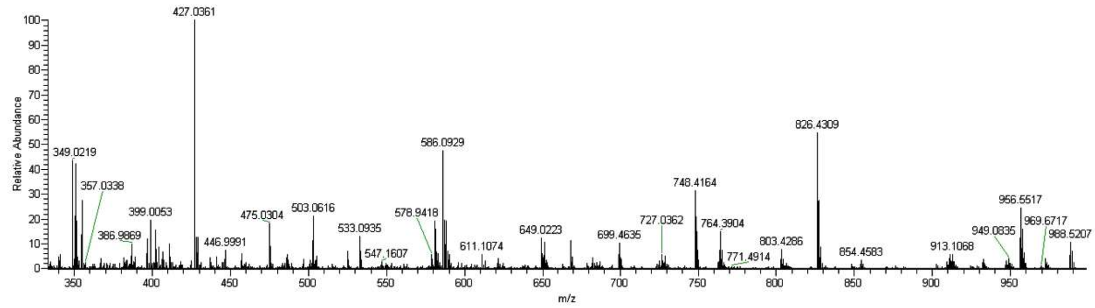


**Figure S9.** MS of complex **1**

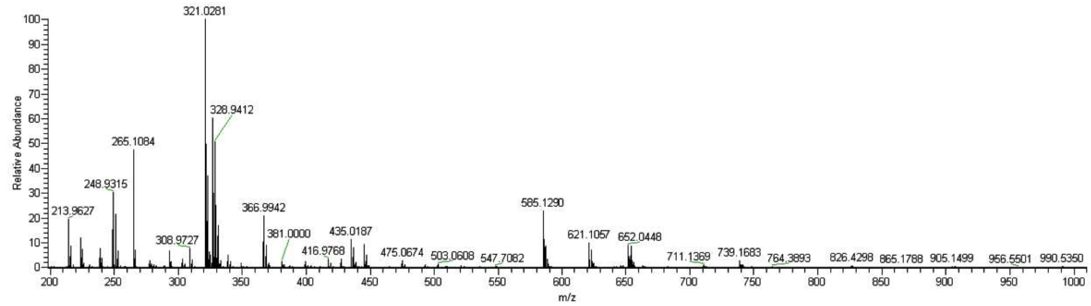


**Figure S10.** MS of complex **2.**

## Experimental methods

**Complex 1 induced $Ca^{2+}$ Fluctuation.** The level of intracellular free $Ca^{2+}$ was decided by using a fluorescent dye Fluo-3 AM which can across the MGC80-3 cell membrane and be cut into Fluo-3 by intracellular esterase. The Fluo-3 can specifically combine with the $Ca^{2+}$ and has a strong fluorescence with an excitation wavelength of 488.0 nm. After exposed to copper(II) complex **1** (10.0 $\mu$M), the MGC80-3 cells were harvested and washed twice with PBS, then resuspended in Fluo-3 AM (5.0 μM) for 30.0 min in dark. Detection of intracellular $Ca^{2+}$ was carried by Flow cytometer at 525.0 nm excitation wavelength.

**Complex 1 induced the loss of Δψ in MGC80-3 cells.** Depolarization of mitochondrial membrane potential (Δψ) for cell apoptosis results in the loss of JC-1 (5,5′,6,6′-tetrachloro-1,1′,3,3′-tetraethylbenzimidazolylcarbocyanine) staining from the mitochondria and a decrease in intracellular fluorescence intensity. After 24.0 h treatment with or without copper complexes **1** (10 μM), the MGC80-3 cells were harvested and washed twice in cold PBS, then resuspended in JC-1 staining (5 μg/mL) for 20-30 min in dark, and examined by flow cytometry.[1–8] The emission fluorescence for JC-1 was monitored at 590 nm,

under the excitation wavelength at 488 nm.

**Assessment on the caspase-3 and caspase-9 activation for MGC80-3 cell apoptosis.** The measurement of caspase-3 and caspase-9 activity was performed by CaspGLOW Fluorescein Active Caspase-3 and caspase-9 Staining Kit.[1–8] $1\times10^6$ of MGC80-3 cells were cultured for 24.0 h. After a treatment with copper complex **1** (10 μM) for 24 h, these MGC80-3 cells were harvested and washed 5 times with cold PBS and were then mixed with 500 μL culture. 1.0 μL of FITC-DEVD-FMK or FITC-LEHD-FMK was consequently added and incubated for 2.0 h at 37 °C with 5% $CO_2$. The MGC80-3 cells were then examined by a FACSAria II flow cytometer equipped with a 488 nm argon laser and results were represented as the percent change on the activity in comparing with the control cells.

**Reactive oxygen species (ROS) levels detection.** DCFH-DA is a freely permeable tracer specific for ROS. DCFH-DA can be deacetylated by intracellular esterase to the non-fluorescent DCFH which is oxidized by ROS to the fluorescent compound 2′,7′-dichloro florescein (DCF). Thus, $1.0\times10^6$ cells were exposed to copper complex **1** (10 μM) for 24 h, and 1.0 mM $H_2O_2$ used as a positive control of ROS production. After the exposure, these MGC80-3 cells were harvested, washed once with ice-cold PBS and incubated with DCFH-DA (100.0 μM in a final concentration) at 37 °C for 20-30 min in the dark. Finally, the MGC80-3 cells were washed again and maintained in 1.0 mL PBS. Finally, the ROS generation was assessed from these cells each sample by FACSAria II flow cytometer with excitation and emission wavelengths of 488.0 and 530.0 nm, respectively.[1–10]

**Western blotting.** The MGC80-3 cells harvested from each well of the culture plates were lysed in 150 μL of extraction buffer consisting of 149.0 μL of RIPA Lysis Buffer and 1.0 μL PMSF (100.0 mM). The suspension was centrifuged at 10000 rpm at 4.0 °C for 10.0 min, and the supernatant (10.0 μL for each sample) was loaded onto 10% polyacrylamide gel and then transferred to a microporous polyvinylidene difluoride (PVDF) membrane. Western blotting was performed using anti-cytochrome c, anti-apaf-1, anti-bax, anti-bcl-2 and anti-β-actin antibody and horseradish peroxidase-conjugated antimouse or antirabbit secondary antibody. Protein bands were visualized using chemiluminescence substrate.

## References


1. Qin, Q.P.; Qin, J.L.; Meng, T.; Yang, G.A.; Wei, Z.Z.; Liu, Y.C.; Liang, H.; Chen, Z.F.; Preparation of 6/8/11-Amino/Chloro-Oxoisoaporphine and Group-10 Metal Complexes and Evaluation of Their in Vitro and in Vivo Antitumor Activity. *Sci. Rep.*, **2016**, *6*, 37644; *doi: 10.1038/srep37644*.
2. Li, Y.L.; Qin, Q.P.; Liu, Y.C.; Chen, Z.F.; Liang, H. A platinum(II) complex of liriodenine from traditional Chinese medicine (TCM): Cell cycle arrest, cell apoptosis induction and telomerase inhibition activity via G-quadruplex DNA stabilization. *J. Inorg. Biochem.*, **2014**, *137*, 12–21. *doi: 10.1016/j.jinorgbio.2014.04.001*
3. Chen, Z.F.; Qin, Q.P.; Qin, J.L.; Liu, Y.C.; Huang, K.B.; Li, Y.L.; Meng, T.; Zhang, G.H.; Peng, Y.; Luo, X.J.; Liang, H. Stabilization of G-Quadruplex DNA, Inhibition of Telomerase Activity, and Tumor Cell Apoptosis by Organoplatinum(II) Complexes with Oxoisoaporphine. *J. Med. Chem.* **2015**, *58*, 2159–2179, *doi:10.1021/jm5012484.*
4. Prokop, A.; Czaplewska, J.A.; Clausen, M.; König, M.; Wild, A.; Thorwirth, R.; Schulze, B.; Babiuch, K.; Pretzel, D.; Schubert U.S. et al. Iridium(III) Complexes of Terpyridine and Terpyridine Analogous Ligands Bearing Sugar Residues and Their in vitro Activity. *Eur. J. Inorg. Chem.***2016**, *21*, 3480–3488.*doi: 10.1002/ejic.201600325*
5. Qin, J.L.; Qin, Q.P.; Wei, Z.Z.; Yu, C.C.; Meng, T.; Wu, C.X.; Liang, Y.L.; Liang, H.; Chen, Z.F. Stabilization of c-myc G-Quadruplex DNA, inhibition of telomerase activity, disruption of mitochondrial functions and tumor cell apoptosis by platinum(II) complex with 9-amino-oxoisoaporphine. *Eur. J. Med. Chem.* **2016**, *124*, 417–427, *doi:10.1016/j.ejmech.2016.08.054.*
6. Qin, Q.P.; Qin, J.L.; Meng, T.; Lin, W.H.; Zhang, C.H.; Wei, Z.Z.; Chen, J.N.; Liu, Y.C.; Liang, H.; Chen, Z.F. High in vivo antitumor activity of cobalt oxoisoaporphine complexes by targeting

G-quadruplex DNA, telomerase and disrupting mitochondrial functions. *Eur. J. Med. Chem.***2016**, *124*, 380−392.*doi: 10.1016/j.ejmech.2016.08.063*

7. Meng, T.; Tang, S.F.; Qin, Q.P.; Liang, Y.L.; Wu, C.X.; Wang, C.Y.; Yan, H.T.; Dong, J.X.; Liu Y.C. Evaluation of the effect of iodine substitution of 8-hydroxyquinoline on its platinum(II) complex: cytotoxicity, cell apoptosis and telomerase inhibition,*Med. Chem. Commun*.**2016**, *7*, 1802−1811.*doi: 10.1039/C6MD00201C*
8. Chou, C.C.; Yang, J.S.; Lu, H.S.; Ip, S.W.; Lo, C.; Wu, C.C.; Lin, J.P.; Tang, N.Y.; Chung, J.G.; Chou, M.J.; et al. Quercetin-mediated cell cycle arrest and apoptosis involving activation of a caspase cascade through the mitochondrial pathway in human breast cancer MCF-7 cells. *Arch. Pharm. Res*.**2010**, *33*, 1181–1191.*doi: 10.1007/s12272-010-0808-y*
9. Carvallo-Chaigneau, F.; Trejo-Solis, C.; Gomez-Ruiz, C.; Rodriguez-Aguilera, E.; Macias-Rosales, L.; Cortes-Barberena, E.; Cedillo-Pelaez, C.; Gracia-Mora, I.; RuizAzuara, L.; Madrid-Marina, V.; Constantino-Casas, F. Casiopeina III-ia induces apoptosis in HCT-15 cells in vitro through caspase-dependent mechanisms and has antitumor effect in vivo. *Biometals* **2008**, *21*, 17–28. *doi:10.1007/s10534-007-9089-4*
10. Hsu, C.W.; Kuo, C.F.; Chuang, S.M.; Hou, M.H. Elucidation of the DNA-interacting properties and anticancer activity of a Ni(II)-coordinated mithramycin dimer complex. *Biometals* **2013**, *26*, 1–12, *doi:10.1007/s10534-012-9589-8.*
